# Endothelial SIRT1 prevents adverse arterial remodeling by facilitating HERC2-mediated degradation of acetylated LKB1

**DOI:** 10.18632/oncotarget.9687

**Published:** 2016-05-29

**Authors:** Bo Bai, Andy W.C. Man, Kangmin Yang, Yumeng Guo, Cheng Xu, Hung-Fat Tse, Weiping Han, Maria Bloksgaard, Jo G.R. De Mey, Paul M. Vanhoutte, Aimin Xu, Yu Wang

**Affiliations:** ^1^ State Key Laboratory of Pharmaceutical Biotechnology and Department of Pharmacology and Pharmacy, The University of Hong Kong, Hong Kong, China; ^2^ Department of Medicine, The University of Hong Kong, Hong Kong, China; ^3^ Singapore Bioimaging Consortium, Agency for Science, Technology and Research (A*STAR), Singapore; ^4^ Department of Cardiovascular and Renal Research, Institute of Molecular Medicine, University of Southern Denmark, Odense, Denmark

**Keywords:** hypertension, blood vessel remodeling, endothelial senescence, SIRT1, LKB1, Gerotarget

## Abstract

Aims-SIRT1 exerts potent activity against cellular senescence and vascular ageing. By decreasing LKB1 protein levels, it promotes the survival and regeneration of endothelial cells. The present study aims to investigate the molecular mechanisms underlying SIRT1-mediated LKB1 degradation for the prevention of vascular ageing.

Methods and Results-Co-immunoprecipitation assay demonstrated that SIRT1, *via* its amino-terminus, binds to the DOC domain of HERC2 [HECT and RLD domain containing E3 ubiquitin protein ligase 2], which then ubiquitinates LKB1 in the nuclear compartment of endothelial cells. Site-directed mutagenesis revealed that acetylation at lysine (K) 64 of LKB1 triggers the formation of SIRT1/HERC2/LKB1 protein complex and subsequent proteasomal degradation. *In vitro* cellular studies suggested that accumulation of acetylated LKB1 in the nucleus leads to endothelial activation, in turn stimulating the proliferation of vascular smooth muscle cells and the production of extracellular matrix proteins. Chromatin immunoprecipitation quantitative PCR confirmed that acetylated LKB1 interacts with and activates *TGFβ1* promoter, which is inhibited by SIRT1. Knocking down either SIRT1 or HERC2 results in an increased association of LKB1 with the positive regulatory elements of *TGFβ1* promoter. In mice without endothelial nitric oxide synthase, selective overexpression of human SIRT1 in endothelium prevents hypertension and age-related adverse arterial remodeling. Lentiviral-mediated knockdown of HERC2 abolishes the beneficial effects of endothelial SIRT1 on both arterial remodeling and arterial blood pressure control.

Conclusion-By downregulating acetylated LKB1 protein *via* HERC2, SIRT1 fine-tunes the crosstalk between endothelial and vascular smooth muscle cells to prevent adverse arterial remodeling and maintain vascular homeostasis.

## INTRODUCTION

Aged arteries are characterized by augmented stiffness and reduced compliance, leading to the elevation of arterial blood pressure and increased risk of cardiovascular diseases [1]. Arteries become stiffer with age due to structural remodeling [2]. In human carotid arteries, the intima-media thickness increases nearly linearly with age, as a result of the proliferation/migration of smooth muscle cell and structural/compositional changes in elastin/collagen fibers [3]. Arterial stiffness is a strong predictor of cardiovascular events and all-cause mortality [4].

Crosstalk between the endothelial and surrounding vascular smooth muscle cells control the process of arterial remodeling [5]. In aged arteries, impaired endothelial function promotes non-compensated and pathological remodeling of the blood vessel wall [6]. With reduced blood flow, atherosclerotic lesions, angioplasty or grafting, arterial remodeling is also triggered by local endothelial dysfunction [7]. Reversing or stabilizing arterial remodeling represents a promising approach for both prevention and treatment of cardiovascular complications caused by hypertension and atherosclerosis [8].

SIRT1 is a NAD-dependent protein deacetylase and the most important member of the anti-ageing protein family of sirtuins [9]. SIRT1 in endothelial cells plays a unique vasoprotective role by regulating a number of proteins including endothelial NO synthase (eNOS), liver kinase B1 (LKB1), p53, NFκB, forkhead box protein O1 (FOXO1) and Notch intracellular domain (NICD) [10–14]. Increased SIRT1 activity prevents endothelial senescence [10, 12], promotes endothelial angiogenesis and migration [15], enhances endothelium-dependent vasodilatation [16], and suppresses vascular inflammation and foam cell formation [17]. These beneficial effects contribute in conjunction to the anti-vascular ageing properties of SIRT1.

In humans, variations of the *SIRT1* gene are associated with increased intima-media thickness of the carotid arteries [18]. In mice, neointima formation is accompanied by a progressive down-regulation of SIRT1 [19]. In cultured endothelial cells, laminar flow increases whereas oscillating flow decreases the expression and activity of SIRT1 [20, 21]. Despite this information, it is unclear whether or not endothelial SIRT1 modulates arterial remodeling, and if so, by what mechanism(s).

The serine/threonine protein kinase LKB1 plays an important role in endothelial senescence and vasculogenesis/arteriogenesis [12, 22, 23]. SIRT1 down-regulates the protein levels of LKB1 in endothelial cells by promoting its ubiquitination and proteasome-mediated degradation [10–12, 24]. The present study tested the hypothesis that endothelial SIRT1 prevents non-compensated and/or pathological arterial remodeling and vascular ageing by regulating the protein stability of LKB1.

## RESULTS

### LKB1 accumulation in endothelial cells promotes the proliferation of vascular smooth muscle cells

**Figure 1:**
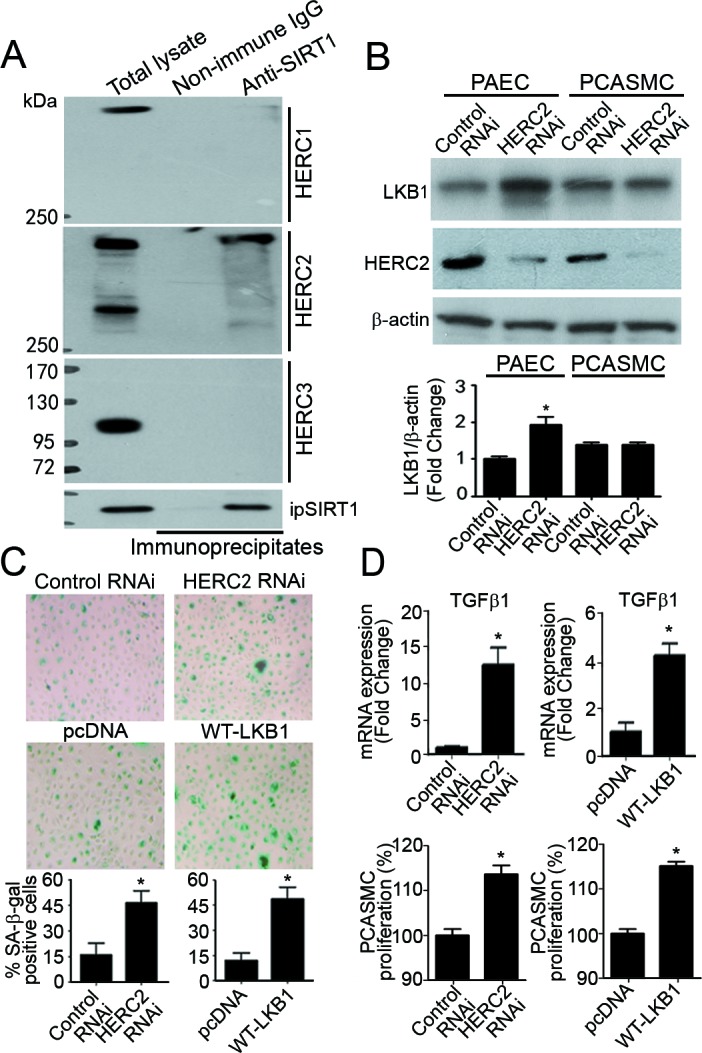
Knocking down HERC2 or overexpressing LKB1 in endothelial cells promotes the proliferation of vascular smooth muscle cells **A.** Co-immunoprecipitation was performed in primary porcine aortic endothelial cells (PAEC) using antibodies against SIRT1. The protein presence of HERC1, HERC2 and HERC3 in the immunoprecipitates were detected by Western blotting using their specific antibodies. **B.** PAEC and porcine coronary artery smooth muscle cells (PCASMC) were transfected with control or HERC2 RNAi. Seventy-two hours after transfection, the protein amount of LKB1 and HERC2 was measured by Western blotting. The results are presented by fold changes against the PAEC control RNAi group. **C.** SA-β-gal staining was performed in PAEC transfected with control RNAi, HERC2 RNAi, pcDNA or LKB1-WT-3FLAG. The positively stained cells were counted manually and the results are expressed as percentage of the total number of cells for comparison. **D.** QPCR was performed to measure the expression of *TGFβ1* in PAEC treated as above (top panels). Results are presented as fold changes against the corresponding treatment control. In addition, the conditioned culture media were collected for proliferation assays (bottom panels). After high speed centrifugation to remove cellular debris, the supernatant was diluted [1:2 ratio] with fresh culture medium and used for incubation with PCASMC for another 36 hours. At the end of treatment, crystal violet staining was performed to compare degrees of cell proliferation. Results are presented as percentage changes. *, *P* < 0.05 *vs* corresponding control groups (*n* = 3-6).

SIRT1 prevents endothelial senescence by enhancing the protein ubiquitination and proteasomal degradation of LKB1 [10, 12, 25]. HERC2, a giant scaffold protein and E3 ubiquitin ligase, is one of the binding partners of SIRT1 [26]. The present results showed that HERC2, but not other members of the HERC family, interacted with SIRT1 (Figure 1A). In endothelial cells derived from murine and porcine arteries, mRNA levels of HERC2 were significantly higher than those of other HERC family members (Supplementary Figure 1). Knocking down HERC2 significantly increased LKB1 protein levels in primary porcine aortic endothelial cells (PAEC), but not in porcine coronary artery smooth muscle cells (PCASMC) (Figure 1B). The portion of senescent cells was significantly augmented to ~45% in PAEC transfected with specific HERC2 RNAi or expression vector encoding LKB1 (Figure 1C).

Quantitative real-time PCR (QPCR) analysis demonstrated that both down-regulation of HERC2 and up-regulation of LKB1 significantly enhanced the mRNA expression levels of transforming growth factor beta-1 (TGFβ1; a morphogen and growth stimulator during vasculogenesis/arteriogenesis [27]) (Figure 1D, top panels). Accordingly, the conditioned media collected from PAEC with decreased HERC2 or increased LKB1 expression significantly stimulated the proliferation of PCASMC (Figure 1D, bottom panels).

**Figure 2:**
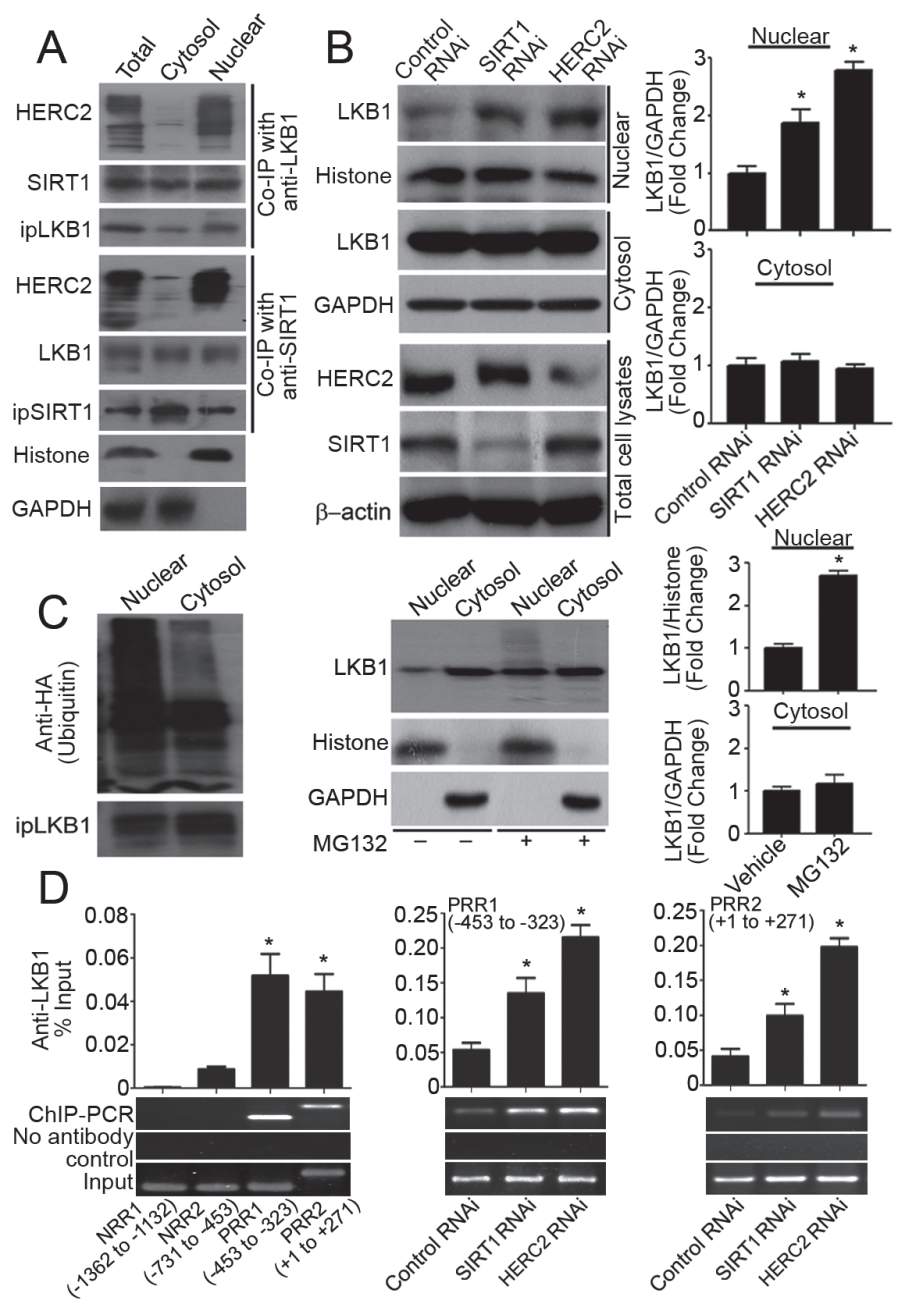
SIRT1 and HERC2 downregulates LKB1 in the nuclear compartments of endothelial cells **A.** Total cell lysates or subcellular (cytosol and nuclear) fractions were prepared from PAEC treated with MG132 (10 μM, six hours) and subjected to co-immunoprecipitation (Co-IP) using antibodies against LKB1 or SIRT1. The amounts of immunoprecipitated HERC2, SIRT1 and LKB1 were detected by Western blotting. GAPDH and histone H3 were probed as markers indicating the purity of the cytosol and nuclear fractions, respectively. **B.** PAEC were transfected with control, SIRT1 or HERC2 RNAi. Seventy-two hours after transfection, nuclear and cytosol fractions were obtained for Western blotting to measure the protein amount of LKB1. Down-regulation of SIRT1 or HERC2 was confirmed in total cell lysates. **C.** PAEC were transfected (left panel) or not (middle and right panels) with an expression vector encoding the HA-tagged ubiquitin (HA-Ub). After treatment with MG132 (10 μM, six hours), cells were collected for immunoprecipitation and Western blotting to probe ubiquitinated LKB1 using anti-HA antibodies (left panel). The protein amount of LKB1 in nuclear and cytosol fractions was measured by Western blotting and are presented as fold changes (middle and right panels). **D.** The binding of LKB1 to *TGFβ1* promoter was evaluated by ChIP-qPCR in human umbilical vein endothelial cells (HUVEC) using primers listed in Supplementary Table 3. After confirming the association of LKB1 to two of the tested regions (left panel), ChIP-qPCR was performed in HUVEC transfected with control, HERC2 or SIRT1 RNAi to compare the association of LKB1 with PRR1 (−453 to −323, middle panel) and PRR2 (+1 to +271, right panel). Bar charts represent the quantitative results by QPCR analysis after normalization against the input DNA. Representative agarose gel images are shown at the bottom. *, *P* < 0.05 *vs* corresponding control groups (*n* = 3-6).

Protein complexes containing HERC2, SIRT1 and LKB1 were detected mainly in the nuclear but not the cytosolic compartment of PAEC (Figure 2A), and human umbilical vein endothelial cells (HUVEC) (Supplementary Figure 2A and 2B). Knocking down either HERC2 or SIRT1 significantly enhanced the accumulation of LKB1 in the nuclear but not the cytosolic fractions of PAEC (Figure 2B). Nuclear LKB1 was heavily ubiquitinated and significantly up-regulated by the treatment with MG132, a specific proteasome inhibitor (Figure 2C).

Next, the association of LKB1 with human *TGFβ1* promoter was evaluated in HUVEC by chromatin immunoprecipitation quantitative PCR (ChIP-qPCR). The region between 453 and 323 base pairs (bp) upstream of the transcriptional start site and a second region from +1 to +271 bp contain positive regulatory activities for cell-specific expression as well as auto-induction by TGFβ [28]. LKB1 bound to both positive regulatory regions of *TGFβ1* promoter, referred to as PRR1 and PRR2, but not to the negative regulatory sequences (NRR1 and NRR2) (Figure 2D, left panel). SIRT1 also associated with PRR1 and PRR2 of *TGFβ1* promoter in HUVEC (Supplementary Figure 2C). Knocking down either HERC2 or SIRT1 significantly enhanced the binding of LKB1 to the positive transcriptional regulatory regions of *TGFβ1* promoter (Figure 2D, middle and right panels).

The above results demonstrate that accumulation of LKB1 in endothelial cells promotes the proliferation of vascular smooth muscle cells. SIRT1 and HERC2 decrease LKB1 protein levels selectively in the nuclear compartment of endothelial cells.

### SIRT1 and HERC2 act synergistically to prevent the nuclear accumulation of LKB1 in endothelial cells

**Figure 3:**
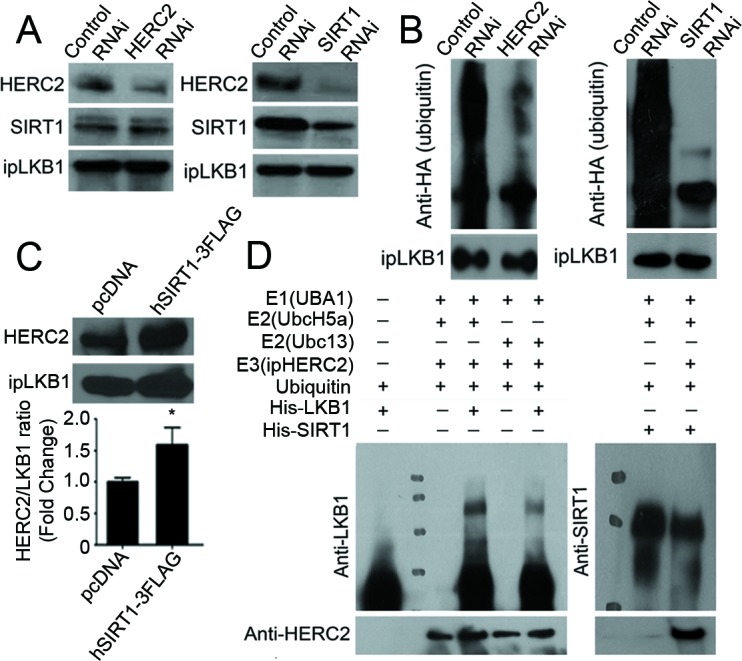
HERC2 mediates the ubiquitination of LKB1 in endothelial cells **A.** Co-immunoprecipitation was performed in PAEC transfected with control, HERC2 or SIRT1 RNAi using antibodies against LKB1. The protein amounts of HERC2 and SIRT1 were measured in the immunoprecipitates by Western blotting using specific antibodies. **B.** PAEC were transfected with HA-Ub together with either control, HERC2 or SIRT1 RNAi. After exposure to MG132 (10 μM, six hours), cells were lysed in a buffer containing 1% SDS and heated for ten minutes at 95°C. Immunoprecipitation was performed with antibody against LKB1. The amount of HA-tagged polyubiquitin in LKB1 was determined by Western blotting using anti-HA antibodies. **C.** PAEC were transiently transfected with pcDNA or hSIRT1-3FLAG for co-immunoprecipitation using antibodies against LKB1. Western blotting was performed to detect HERC2 in the immunocomplexes. The ratio between immunoprecipitated HERC2 and LKB1 was calculated for comparison. *, *P* < 0.05 *vs* pcDNA (*n* = 3). **D.** An *in vitro* ubiqutination assay was performed as described in Methods. The reaction mixture was subjected to Western blotting for detecting the ubiquitinated LKB1 (left) or SIRT1 (right) with antibodies against LKB1 or SIRT1, respectively. When compared to the control sample, ubiquitinated LKB1 appeared as a higher molecular weight species.

Knocking down HERC2 did not inhibit the interactions between SIRT1 and LKB1, but reduced the amount of ubiquitin conjugated to LKB1 (Figure 3, left panels of A and B). By contrast, down-regulation of SIRT1 abolished the interactions between HERC2 and LKB1 as well as the ubiquitination of LKB1 (Figure 3, right panels of A and B). In cells overexpressing SIRT1, the protein interactions between endogenous HERC2 and LKB1 were enhanced significantly (Figure 3C). However, the expression and ubiquitination of SIRT1 were not significantly altered by down-regulation of HERC2, and the protein levels of HERC2 remained stable after transient overexpression of either SIRT1 or LKB1 (Supplementary Figure 3). An *in vitro* ubiquitination assay was performed using immunoprecipitated HERC2, with His-tagged recombinant LKB1 or SIRT1 as the substrates. In the presence of E1 (UBA1) and E2 (UbcH5a or Ubc13), HERC2 was able to ubiquitinate LKB1 but not SIRT1 (Figure 3D).

**Figure 4:**
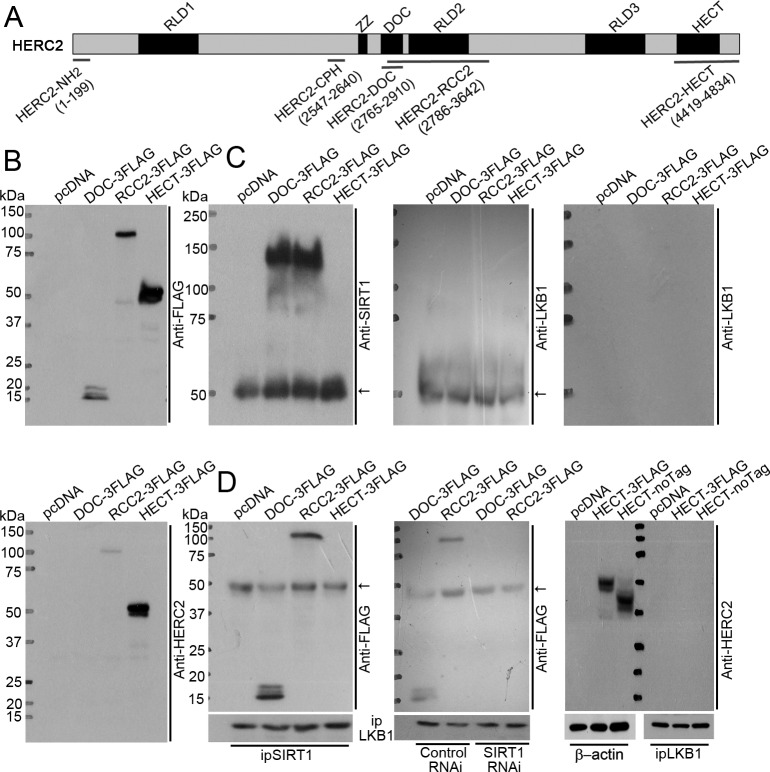
SIRT1 binds to HERC2-DOC domain to facilitate the interactions between HERC2 and LKB1 **A.** HERC2 is a large protein containing three RCC-like domains (RLD 1-3), a zinc finger domain (ZZ), an anaphase-promoting complex homolog domain (DOC) and a carboxy-terminal HECT domain [55]. Two prokaryotic expressing vectors producing HERC2-NH_2_ (amino acid 1-199) and HERC2-CPH (amino acid 2547-2640) fragments (Supplementary Figure 5), and three mammalian expressing vectors encoding HERC2-DOC (amino acid 2765-2910), HERC2-RCC2 (amino acid 2786-3642) and HERC2-HECT (amino acid 4419-4834) domains were used for domain-interaction studies. **B.** HEK293 cells were transiently transfected with pcDNA, DOC-3FLAG, RCC2-3FLAG or HECT-3FLAG. The expressions of HERC2-DOC, HERC2-RCC2 and HERC2-HECT were confirmed by Western blotting using anti-FLAG (top panel) or anti-HERC2 (bottom panel) antibodies, the latter recognizing the carboxyl terminus of HERC2. **C.** Co-immunoprecipitation was performed in lysates from the transfected cells using anti-FLAG antibodies. The eluted immunocomplexes were subjected to Western blotting to detect the presence of SIRT1 (left panel) or LKB1 (middle and right panels) using their specific antibodies. Note that LKB1 was absent after probing with secondary antibodies recognizing both heavy and light chains (middle panel) or only the F(ab) fragment of IgG (right panel). **D.** Co-immunoprecipitation was performed using anti-SIRT1 antibodies. The presence of HERC2 sub-domains was detected by Western blotting using an anti-FLAG antibody (left panel). Interactions between LKB1 and HERC2 domains were investigated by co-immunoprecipitation in cells overexpressing HERC2-DOC or HERC2-RCC2, with co-transfection of control or SIRT1 RNAi (middle panel). In addition, cells overexpressing HERC2-HECT in either FLAG-tagged or non-tagged form were subjected to co-immunoprecipitation using anti-LKB1 antibodies. The presence of HERC2 sub-domains was examined by Western blotting using anti-HERC2 antibodies (right panel). The arrows indicate non-specific signals from IgG heavy chain.

HERC2 protein consists of three RCC-like domains (RLD1-3), a zinc finger domain (ZZ), an anaphase-promoting complex subunit 10/DOC domain, and a carboxy-terminal HECT domain (Figure 4A). Next, mammalian expression vectors were constructed to produce the FLAG-tagged DOC (HERC2-DOC) and RCC2 (HERC2-RCC2) domains, which are uniquely present in HERC2 but not the other HERC family members [29], as well as the FLAG-tagged HECT domain (HERC2-HECT) that possesses E3 ubiquitin-ligase activity and shares high sequence homology with HERC3, HERC4, HERC5 and HERC6 [30, 31]. Expression of the three domains of HERC2 was confirmed in transiently transfected HEK293 cells by Western blotting using antibodies recognizing the FLAG epitope or the carboxyl-terminus of HERC2 (Figure 4B).

Next, co-immunoprecipitations were performed to elucidate domain-domain interactions. Endogenous SIRT1 was immunoprecipitated (with anti-FLAG antibodies) from cells overexpressing HERC2-DOC or HERC2-RCC2, but not HERC2-HECT domains (Figure 4C, left panel). LKB1 could not be detected in any of the immunocomplexes by Western blotting using anti-LKB1 and secondary antibodies recognizing both heavy and light chains or only the F(ab) fragment of IgG (Figure 4C, middle and right panels). When using anti-SIRT1 antibodies for co-immunoprecipitation, HERC2-DOC and HERC2-RCC2, but not HERC2-HECT, were present in the immunocomplexes (Figure 4D, left panel). When using anti-LKB1 antibodies for co-immunoprecipitation, HERC2-DOC and HERC2-RCC2 were barely detected in the immunocomplexes by anti-FLAG antibodies (Figure 4D, middle panel). The weak interactions between these two domains and LKB1 were abolished after knocking down SIRT1 (Figure 4D, middle panel). In cells overexpressing HERC2-HECT with or without a FLAG-tag, co-immunoprecipitation performed with anti-LKB1 antibodies could not precipitate any HERC2 subdomains (Figure 4D, right panel).

In line with the above findings, overexpression of HERC2-HECT did not reduce the amount of LKB1 protein and had no effects on the ubiquitination levels of LKB1 or SIRT1 (Supplementary Figure 4). *In vitro* pull-down experiments showed that the amino-terminus (HERC2-NH_2_) and the CPH domain (HERC2-CPH) of HERC2 did not bind to SIRT1 or LKB1 (Supplementary Figure 5A), whereas the p53 protein was precipitated by HERC2-CPH [32]. Compared to full-length SIRT1, a truncated form of SIRT1 lacking the amino-terminal 105 residues could not downregulate LKB1, and lost the binding capacity to HERC2 (Supplementary Figure 5B and 5C).

The above findings indicate that SIRT1 *via* its amino-terminus binds to the HERC2-DOC domain and brings HERC2 in close proximity to LKB1 (Supplementary Figure 5D). The formation of the SIRT1/HERC2/LKB1 protein complex triggers subsequent ubiquitination and proteasomal degradation of LKB1 in the nuclear compartment of endothelial cells.

### Acetylation of LKB1 at K64 facilitates the formation of protein complexes with SIRT1 and HERC2

**Figure 5:**
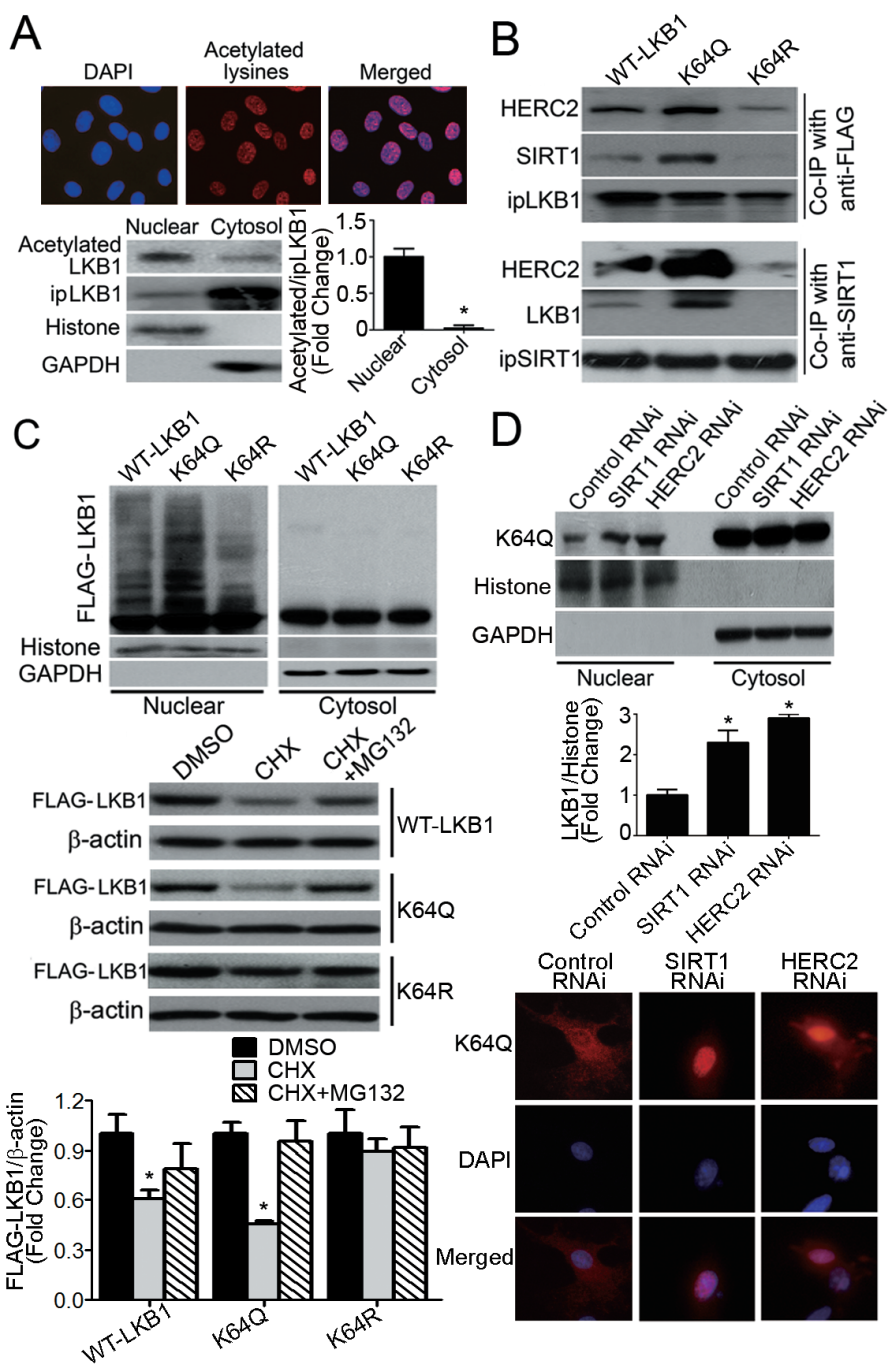
Acetylation of LKB1 at lysine 64 facilitates its interactions with SIRT1/HERC2 and protein degradation in the nuclear compartment of endothelial cells **A.** PAEC were subjected to immunofluorescence staining with antibodies against acetylated-lysine (top panel). Magnification, 400x. LKB1 was immunoprecipitated from nuclear or cytosol fractions of PAEC. The amount of acetylated LKB1 was measured by Western blotting using antibodies against acetylated-lysine (bottom panel). The ratio between acetylated and immunoprecipitated (ip) LKB1 was calculated for comparison. *, *P* < 0.05 *vs* nuclear fractions (*n* = 3). **B.** Co-immunoprecipitation (Co-IP) was performed in PAEC transiently overexpressing FLAG-tagged WT-LKB1, K64Q or K64R, using either anti-FLAG or anti-SIRT1, followed by Western blotting with specific antibodies. **C.** PAEC were co-transfected with HA-Ub plus vectors expressing WT-LKB1, K64Q or K64R. After treatment with MG132 (10 μM, six hours), the nuclear and cytosol fractions were collected for Western blotting to evaluate the presence of LKB1 with attached polyubiquitin chains (top panel). Histone H3 and GAPDH were probed as markers for the nuclear and cytosol fractions, respectively. PAEC overexpressing WT-LKB1, K64Q or K64R were treated or not with cycloheximide (CHX, 50 μg/ml), in the absence or presence of MG132 (10 μM), for six hours. The protein amounts of WT-LKB1, K64Q and K64R were quantified by Western blotting using anti-FLAG antibodies (bottom panel). *, *P* < 0.05 *vs* DMSO-treated samples (*n* = 3). D, Cells overexpressing K64Q were transfected with control, SIRT1 or HERC2 RNAi and subjected to subcellular fractionation to obtain the nuclear and cytosol fractions. The amount of K64Q was measured by Western blotting (top panel) and the results calculated as the ratio between LKB1 and GAPDH for cytosol fractions (data not shown), or LKB1 and histone for nuclear fractions (middle panel). *, *P* < 0.05 *vs* control RNAi (*n* = 3). The cellular distribution of K64Q was analyzed by immunofluorescence staining using anti-FLAG antibodies (bottom panel).

In PAEC, proteins containing acetylated-lysine residues mainly accumulated in the nuclei (Figure 5A, top panels). Likewise, the majority of LKB1 within the nuclear compartment was acetylated (Figure 5A, bottom panels). To address whether or not acetylation plays a role in the ubiquitination and degradation of LKB1, a series of single, double or triple mutants with lysine (K) residues 48, 62, 64 and/or 329 replaced by glutamine (Q, as a mimic of acetyl-lysine) or arginine (R, as a mimic of non-acetylated lysine) were examined (data not shown). K64Q significantly enhanced the binding of LKB1 to SIRT1/HERC2, whereas the K64R mutation attenuated the interaction between LKB1 and SIRT1, and the subsequent association with HERC2 (Figure 5B). The increased interaction between K64Q and SIRT1 was further confirmed by an *in vitro* pull-down assay (Supplementary Figure 6A). Compared to WT-LKB1 and K64Q, K64R exhibited an enhanced interaction with STRADα (Supplementary Figure 6B), a pseudokinase that inhibits LKB1 nuclear importation [33]. As a result, K64R protein levels were significantly decreased in the nucleus of endothelial cells (Supplementary Figure 6C).

When compared to wild type (WT)-LKB1 and K64R, K64Q was heavily ubiquitinated in the nuclear compartment of PAEC (Figure 5C, top panel). In the presence of a protein synthesis inhibitor, cycloheximide, K64Q exhibited significantly decreased protein stability, whereas K64R remained stable (Figure 5C, bottom panel). Knocking down either SIRT1 or HERC2 significantly increased K64Q protein levels in the nuclear but not the cytosolic fractions of PAEC (Figure 5D). Consistently, overexpression of SIRT1 primarily reduced the nuclear content of K64Q (Supplementary Figure 7).

**Figure 6:**
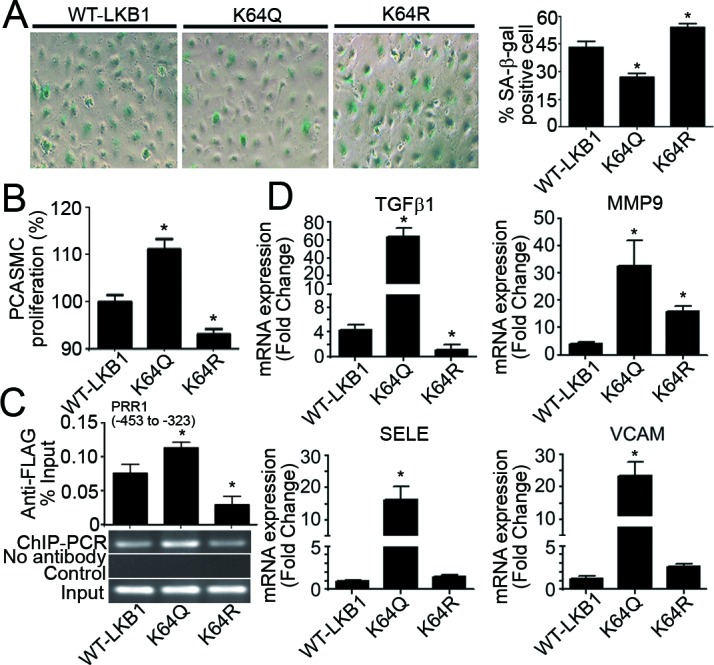
Overexpression of K64Q causes endothelial activation and promotes vascular smooth muscle cell proliferation **A.** SA-β-gal staining was performed in PAEC transfected with vectors encoding WT-LKB1, K64Q or K64R. Positively stained cells were counted manually and the results expressed as percentage of the total number of cells for comparison. **B.** Forty-eight hours after transfection, the conditioned culture media were collected for incubation with PCASMC. After 36 hours of treatment, crystal violet staining was performed to evaluate PCASMC proliferation. Results are presented as percentage changes compared to the WT-LKB1 group. **C.** ChIP-qPCR was performed in HUVEC overexpressing WT-LKB1, K64Q or K64R using an anti-FLAG antibody to measure their associations with the PRR1 (−453 to −323) of human *TGFβ1* promoter. **D.** QPCR was performed to measure the gene expressions in PAEC overexpressing WT-LKB1, K64Q or K64R. *, *P* < 0.05 *vs* corresponding control groups (*n* = 3-6).

Compared to WT-LKB1, overexpression of K64Q for 48 hours reduced the total number of PAEC by approximately 32%, due largely to the induction of apoptosis (Supplementary Figure 8). Accordingly, the percentage staining of senescence-associated β-galactosidase (*SA-β-gal) in* PAEC overexpressing K64Q was only ~25% (Figure 6A). Conditioned media from PAEC overexpressing K64Q stimulated PCASMC proliferation to a significantly higher level than those collected from cells overexpressing WT-LKB1 or K64R (Figure 6B). Moreover, the amount of K64Q associated with TGFβ1 promoter was significantly increased when compared to WT-LKB1 and K64R (Figure 6C). In addition to TGFβ1, overexpression of K64Q significantly enhanced the mRNA expressions of other genes involved in endothelial activation and extracellular matrix deposition (Figure 6D).

Taken in conjunction, these results reveal that acetylation of K64 plays a pivotal role in determining the protein stability and function of LKB1 in endothelial cells, in turn modulating the proliferation of vascular smooth muscle cells.

### Knocking down HERC2 abolishes the vascular protective effects of endothelial SIRT1 in eNOS-deficient mice

**Figure 7:**
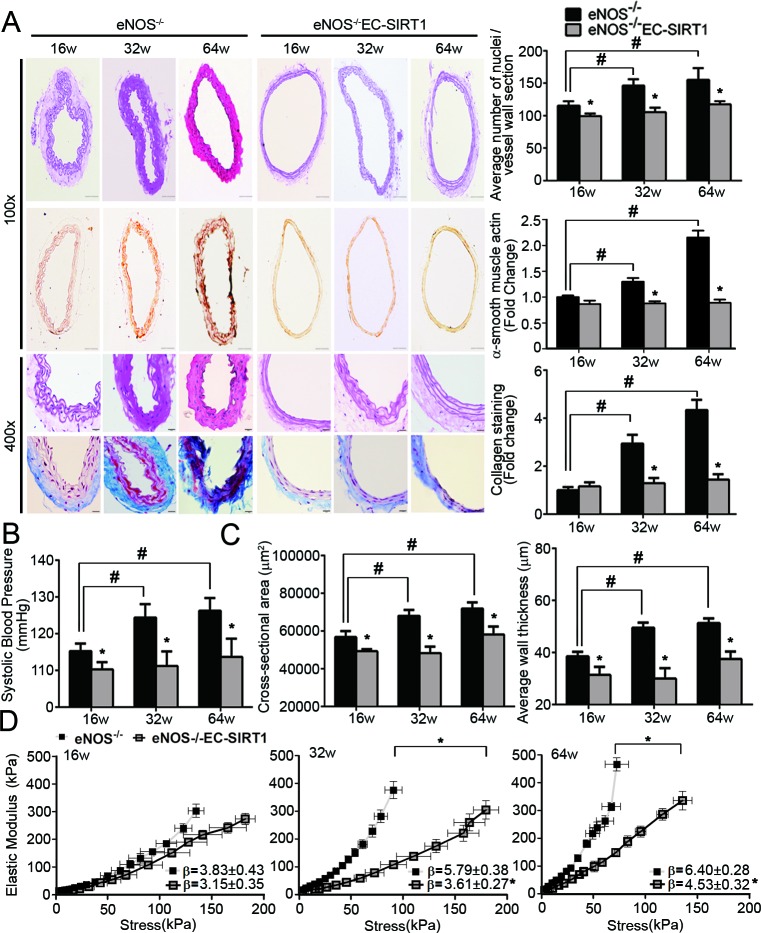
Endothelial overexpression of SIRT1 prevents adverse arterial remodeling and alleviates hypertension in eNOS-deficient mice **A.** The structural properties of carotid arteries collected from 16-, 32-, and 64-weeks old eNOS^−/−^ or eNOS^−/−^EC-SIRT1 mice were analyzed by H&E, α-smooth muscle actin or Masson Trichrome staining (left panels). Magnification, 100x and 400x. The total numbers of nuclei were counted manually in ten sections of each group of mice for comparing cellularity (right top panel). The area of positively stained α-smooth muscle actin (right middle panel) and the pixels of collagen deposition (right bottom panel) were quantified using ImageJ software for comparison. **B.** Arterial systolic blood pressure was recorded using the tail-cuff method (see Supporting Information) in mice of different ages for comparison. **C.** Pressure myography was performed to compare the passive geometries and mechanical properties of the vascular wall in carotid arteries collected from eNOS^−/−^ and eNOS^−/−^EC-SIRT1 mice at different ages. Wall thickness and cross-sectional areas at a distending pressure of 110 mmHg were recorded for comparison. **D.** Young's elastic modulus were calculated by fitting the circumferential stress-strain data to an exponential curve as described in Methods. The results are presented as a function of stress. *, *P* < 0.05 *vs* age matched eNOS^−/−^ mice; #, *P* < 0.05 *vs* 16-weeks old eNOS^−/−^ mice (*n* = 5-8).

The pathophysiological significance of the above findings was examined in eNOS-deficient mice without (eNOS^−/−^) or with (eNOS^−/−^EC-SIRT1) endothelial overexpression of human SIRT1. The protein amount of SIRT1 was significantly decreased with age in carotid arteries of eNOS^−/−^, but maintained at a higher level in those of eNOS^−/−^EC-SIRT1 mice (Supplementary Figure 9A). While HERC2 expression was not significantly different, LKB1 protein levels increased in carotid arteries of aged eNOS^−/−^ mice. Compared to eNOS^−/−^ mice, the age-induced accumulation of acetylated LKB1 was significantly attenuated in carotid arteries of eNOS^−/−^EC-SIRT1 mice (Supplementary Figure 9B). The cellularity of common carotid arteries from eNOS^−/−^EC-SIRT1 mice was significantly decreased, as revealed by both manual counting the number of nuclei and histological staining of α-smooth muscle actin (Figure 7A). Moreover, endothelial overexpression of SIRT1 prevented the age-related progressive deposition of collagen in carotid arteries of eNOS^−/−^ mice (Figure 7A). The arterial blood pressure of eNOS^−/−^EC-SIRT1 mice was significantly lower than that of eNOS^−/−^ mice from different age groups (Figure 7B).

The structural and mechanical properties of carotid arteries were further evaluated by using a pressure myograph. The cross-sectional area and average thickness of the blood vessel wall (measured at a transmural pressure of 110 mmHg) were significantly reduced in preparations of eNOS^−/−^EC-SIRT1 compared to those of eNOS^−/−^ mice (Figure 7C). With age, the elastic modulus-stress curves in preparations of eNOS^−/−^ mice were shifted significantly leftward when compared to those of eNOS^−/−^EC-SIRT1 arteries (Figure 7D), indicating that endothelial overexpression of SIRT1 prevented age-induced vascular stiffness in carotid arteries of eNOS^−/−^ mice.

**Figure 8:**
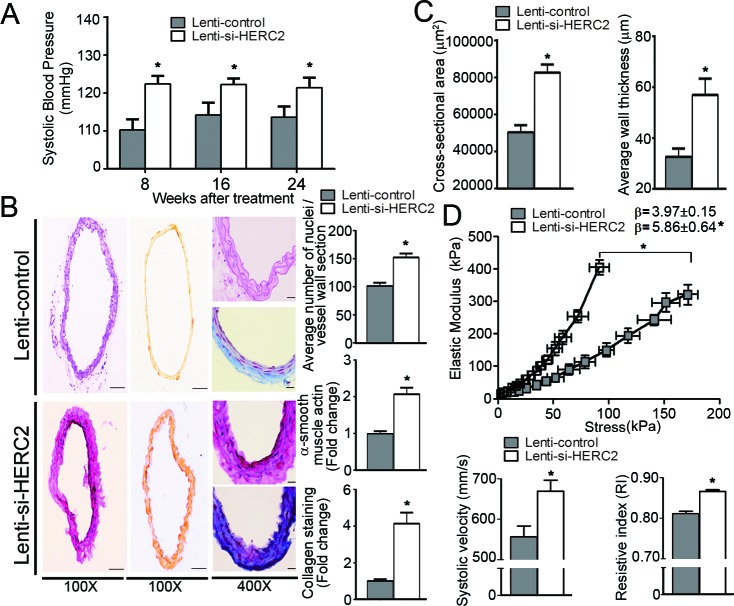
Lentiviral-mediated knockdown of HERC2 abolishes the vascular protective effects of endothelial SIRT1 in eNOS-deficient mice **A.** Arterial systolic blood pressure was recorded using a tail-cuff system in eNOS^−/−^EC-SIRT1 mice treated with Lenti-control or Lenti-si-HERC2, at eight-, 16- and 24-weeks after viral injections. **B.** The carotid arteries were collected from eNOS^−/−^EC-SIRT1 mice after 24-weeks of treatment with Lenti-control or Lenti-si-HERC2 for analyzing the blood vessel wall cellularity and collagen deposition as in Figure 7A. **C.** Pressure myography was performed to compare the passive geometries and mechanical properties of the blood vessel wall in carotid arteries collected from eNOS^−/−^EC-SIRT1 mice after 24-weeks of treatment with either Lenti-control or Lenti-si-HERC2. Wall thickness and cross-sectional areas were recorded as in Figure 7C for comparison. **D.** The β values and Young's elastic modulus were calculated by fitting the circumferential stress-strain data to an exponential curve as described in Methods. The results are presented as a function of stress (top panel). Noninvasive measurement of velocity profiles in right common carotid arteries was performed using pulsed Doppler ultrasound with Vevo 2100 imaging system (see Supporting Information) (bottom panel). The peak systolic velocity and resistive index were recorded for comparison. *, *P* < 0.05 *vs* eNOS^−/−^EC-SIRT1 mice treated with Lenti-control (*n* = 5-6).

Next, control piLenti-siRNA lentivirus (Lenti-control) or piLenti-HERC2siRNA-GFP lentivirus (Lenti-si-HERC2; for knocking down murine HERC2) were administered into eNOS^−/−^EC-SIRT1 mice. Western blotting confirmed that HERC2 was significantly down-regulated, whereas the protein levels of LKB1 and TGFβ1 augmented in carotid arteries of eNOS^−/−^EC-SIRT1 mice after treatment with Lenti-si-HERC2 (Supplementary Figure 10A). As early as eight-weeks after viral treatment, the systolic blood pressure was significantly augmented by knocking down HERC2 (Figure 8A). After 24-weeks of viral treatment, knocking down HERC2 significantly increased the cellularity and collagen deposition in carotid arteries of eNOS^−/−^EC-SIRT1 mice (Figure 8B), which also exhibited significantly elevated expression of *TGFβ1, MMP9, Collagen I and III* (Supplementary Figure 10B). The cross-sectional area and blood vessel wall thickness were augmented significantly by knocking down HERC2 in eNOS^−/−^EC-SIRT1 mice (Figure 8C). Treatment with Lenti-si-HERC2 increased the β-value (5.86±0.64 vs 3.97±0.15) of the stress-strain curves in arteries of eNOS^−/−^EC-SIRT1 mice, in line with the significantly leftward shifted elastic modulus-stress curves (Figure 8D, top panel). Noninvasive measurement of blood flow by echo ultrasonography suggested a significantly increased peak systolic velocity and resistive index in the right common carotid arteries of eNOS^−/−^EC-SIRT1 mice treated with Lenti-si-HERC2, compared to those of the Lenti-control groups (Figure 8D, bottom panels).

Collectively, these results demonstrate that down-regulation of HERC2 abolishes the beneficial effects of endothelial SIRT1 on the prevention of adverse arterial remodeling and hyperplasia in eNOS-deficient mice.

## DISCUSSION

Arterial remodeling is a physiological process that involves the growth, death, migration of cells, and the synthesis, deposition, degradation of extracellular matrix. Arterial stiffness due to non-compensated remodeling contributes to the high prevalence of hypertension and cardiovascular events in aged populations [1]. Effective control of arterial blood pressure requires a concomitant reduction of large artery stiffness [34]. Among the sirtuin family, SIRT1 is the most conserved member with diversified anti-ageing and vasoprotective properties [24, 35, 36]. The present findings demonstrate that formation of SIRT1/HERC2/LKB1 complex in endothelial cells controls the process of arterial remodeling and represents a potential target for the development of de-stiffening drugs.

HERC2 and LKB1 independently bind to different regions of SIRT1, which facilitates the formation of SIRT1/HERC2/LKB1 protein complexes. The amino-terminus of SIRT1 interacts with HERC2-DOC, a ~125 amino acid sub-region of HERC2 that is not involved in the binding with its other protein substrates, including XPA [37], BRCA1 [30], p53 [32], and RNF8 [38]. The binding with LKB1 does not involve the amino-terminus of SIRT1. However, a truncated form of SIRT1 without the amino-terminus cannot down-regulate LKB1. The stoichiometry of the SIRT1/HERC2/LKB1 complex explains the observations that knocking down HERC2 does not affect the interaction between SIRT1 and LKB1, whereas knocking down SIRT1 abolishes that of LKB1 with HERC2. Thus in aged arteries, although HERC2 levels remain stable, the amount of acetylated LKB1 rises due to a significant reduction in SIRT1.

LKB1 in endothelial cells regulates the early stages of embryonic vascular remodeling. Embryonic lethality caused by vascular defects is a common phenotype in mice with whole body homozygous ablation of *LKB1* [22], endothelium-restricted deletion of *LKB1* [23], or partial endothelial-deletion of *LKB1* [39, 40]. In matured blood vessels, endothelial overexpression of LKB1 inhibits VEGF signaling [41], enhances senescence [10, 12], prevents proliferation and induces apoptosis [42]. Here, the results suggest that in aged arteries, accumulation of acetylated LKB1 in the endothelium stimulates the proliferation of surrounding vascular smooth muscle cells and triggers adverse arterial remodeling.

TGFβ1 is causally involved in post-angioplasty restenosis, post-infarction myocardial remodeling as well as other fibrotic disorders [43]. Increased TGFβ1 signaling is associated with elastic fiber disruption and hypertensive phenotypes in genetically modified mice models. Positive correlations exist between TGFβ1 expression and LKB1 function [23, 44, 45]. However, detailed information about the molecular mechanisms underlying LKB1-mediated TGFβ expressions is lacking. The present study demonstrates that acetylated LKB1 acts as a transcriptional activator of *TGFβ1* by binding to the cis-elements of its promoter. SIRT1 prevents the interactions of LKB1 with *TGFβ1* promoter, by facilitating HERC2-mediated ubiquitination and proteasomal degradation of acetylated LKB1 in the nuclei of endothelial cells.

Lysine acetylation represents a key mechanism to regulate LKB1 protein stability and function. LKB1 is acetylated in the liver and white adipose tissues, and such acetylation is enhanced by high glucose levels [46]. Starvation decreases LKB1 acetylation and increases its kinase activity to improve glucose tolerance [47]. Loss-of-SIRT1 in senescent endothelial cells leads to the accumulation of LKB1 mainly in its acetylated form [12]. The present study has extended these previous findings and reveals that nuclear LKB1 is mainly in the acetylated form and represents a major portion highly sensitive to SIRT1-mediated proteasomal degradation, which may not involve the chemical reaction of deacetylation. Unlike the acetylated histone H3 peptide, incubation with SIRT1 protein does not remove the acetyl group attached to K64 of a synthetic LKB1 peptide (Supplementary Figure 11). The protein stabilities of other SIRT1 targets, including NICD [13], p53 [48, 49], NFκB [50], and FOXO1 [51], are also negatively correlated with their acetylation levels. It is possible that SIRT1 mediates the degradation of acetylated protein targets in a cell-specific manner by forming different protein complexes that contain distinct E3 ligases.

The present animal studies reveal that endothelial overexpression of SIRT1 prevents adverse vascular remodeling and hypertension in eNOS-deficient mice. In particular, increased SIRT1 levels in the endothelium prevent excessive proliferation of vascular smooth muscle cells and deposition of collagen, thus enhancing blood vessel compliance and antagonizing age-induced arterial stiffness. Down-regulation of HERC2 abolishes the beneficial effects of endothelial SIRT1 overexpression on arterial remodeling and blood pressure control. The present findings support a key role of HERC2 in SIRT1-mediated vasoprotection, by promoting the proteasomal degradation of acetylated LKB1 and preventing its accumulation in the endothelium of aged arteries.

Based on the above findings, it is proposed that the levels of acetylated LKB1 protein augment in endothelial cells under conditions such as nutritional imbalance or disruption of the circadian rhythm, which leads to an increased production of the intermediary metabolite acetyl-CoA [52]. Upon acetylation at K64, LKB1 exhibits decreased binding to STRADα but enhanced interaction with SIRT1. The complex formation between acetylated LKB1 and SIRT1/HERC2 triggers the nuclear degradation of the former in endothelial cells. Thus, timely formation of SIRT1/HERC2/LKB1 complex in endothelial cells allow an efficient adaptation to the dynamic changes of blood constituents (e.g. nutrients, metabolites and oxidative radicals). In senescent endothelial cells or aged arteries, loss-of-SIRT1 expression or function results in an increased nuclear accumulation of acetylated LKB1, leading to irreversible structural alterations of the blood vessel wall, adverse arterial remodeling and vascular stiffness.

## MATERIALS AND METHODS

### Animals

All animal care and experimental procedures (details in online Supplementary file) were approved by the Committee on the Use of Live Animals for Teaching and Research of the University of Hong Kong, and carried out in accordance with the Guide for the Care and Use of Laboratory Animals published by the US National Institutes of Health (Eighth Edition, National Research Council, USA, 2011).

### RNA interference

Three sets of small interfering RNAs (RNAi) targeting human *HERC2* (GCAACACAGTCGTGAAAGA; GCGGAAGCCTCATTAGAAA; and GAGCTGATTTCTTGAGTAA) or porcine *HERC2* (GGACTTTCTGTGTCAAATA; CATTAAGACACATCAAGAA and GGAGGATTGCTCAGAAGAT) and two sets of RNAi (GGCACAGATCCTCGAACAA and CCAGTAGCACTAATTCCAA) targeting the conserved sequences of human and porcine *SIRT1* were purchased from RiboBio (Guangzhou, China). The mixture containing 50 nM RNAi was used for transfection using Lipofectamine 2000 (Life Technologies, Grand Island, NY, USA).

Four sets of piLenti-siRNA-GFP lentiviral vectors targeting murine *HERC2* were obtained from Applied Biological Materials Inc and tested in 3T3-L1 cell cultures (data not shown). The most effective siRNA (ACAGAGACTGTTACCTATTAAACCCTGCC) was packaged to produce the recombinant lentivirus (piLenti-HERC2siRNA-GFP) with a titer of 1.6 × 10^8^ IU/ml for subsequent administration to mice.

### Plasmids and site-directed mutagenesis

The mammalian expression vectors hSIRT1-3FLAG, pcDNA-SIRT1 and ∆SIRT1-FLAG were used for overexpressing full-length human SIRT1 with a carboxy-terminal triple-FLAG tag [10], full-length human SIRT1 without any fusion tags [26], and a truncated FLAG-tagged human SIRT1 lacking 105 amino acids at the amino-terminus, respectively.

Primers (Supplementary Table 1) were designed by referring to the cDNA sequence of human HERC2 (NM_004667.5) for cloning three gene fragments into the pCMV-3Tag-3 mammalian expression vector (Agilent Technologies, La Jolla, CA, USA). DNA sequencing confirmed that the three vectors, including DOC-3FLAG, RCC2-3FLAG, and HECT-3FLAG, encode the sub-domains of HERC2 from amino acid 2765 to 2910 (HERC2-DOC), 2786 to 3642 (HERC2-RCC2) and 4419 to 4834 (HERC2-HECT), respectively, all of which contain a carboxy-terminal triple-FLAG tag. In addition, an expression vector (HECT-noTag) encoding the HERC2-HECT domain without any fusion tags was constructed.

The cDNA of murine LKB1 (NM_011492.4) was sub-cloned into the pCMV-3Tag-3 vector using forward (5′-TGGTACCGAGCTCGGATCCACTAGTCCAGTGT-3′) and reverse (5′-ATATTTCTCGAGTCGCTGCTGCTTGCA-3′) primers to construct a mammalian expression vector (LKB1-WT-3FLAG) encoding full-length LKB1 with a carboxy-terminal triple-FLAG tag. Site-directed mutagenesis was performed using LKB1-WT-3FLAG as the template to obtain vectors encoding LKB1 mutants with the lysine (K) 64 residue replaced by either arginine (R) (LKB1-K64R-3FLAG) or glutamine (Q) (LKB1-K64Q-3FLAG). All mutations were confirmed by DNA sequencing.

The prokaryotic vectors for expressing the amino-terminus (HERC2-NH_2_, residues 1–199) and CPH domain (HERC2-CPH, residues 2547–2640) of human HERC2 as fusion proteins with glutathione S-transferase were kindly provided by Dr José Luis Rosa [32]. The prokaryotic vectors expressing polyhistidine (His)-tagged human SIRT1 (pPROEX-HTb-SIRT1) or murine LKB1 (pPROEX-HTb-LKB1) were constructed by sub-cloning the cDNAs (from hSIRT1-3FLAG and LKB1-WT-3FLAG, respectively) into a pPROEX-HTb vector (Life Technologies).

### *In vitro* ubiquitination assay

HERC2 protein was immunoprecipitated from MDA-MB-231 cell lysates for assaying ubiquitination *in vitro*. In brief, 100 ng ubiquitin-activating enzyme (E1), 200 ng ubiquitin-conjugating enzyme (E2), one μg FLAG-tagged ubiquitin, and two μg purified His-tagged LKB1 or SIRT1 protein as substrates were included in an assay buffer containing 25 mM HEPES (pH 7.4), 10 mM NaCl, 10 mM MgCl_2_, 1 mM EGTA, 0.5 mM dithiothreitol and 3 mM ATP. The reaction was performed at 37°C for one hour. Ubiquitinated-LKB1 or SIRT1 was detected by Western blotting using anti-LKB1 or anti-SIRT1antibodies, respectively.

### Microscopic morphometry and vessel wall cellularity

The common carotid artery was carefully removed, fixed in neutral buffered formaldehyde, dehydrated and embedded in paraffin. The tissue blocks were cut into sections of five μm thickness for hematoxylin and eosin (H&E) staining, immunohistochemistry or the evaluation of collagen deposition using a Trichrome Stain (Masson) Kit (Sigma, Saint Louis, MO, USA). The intimal (area between the lumen and the internal elastic lamina), medial (area between the internal and the external elastic lamina), and total blood vessel wall area were determined by computerized digital planimetry, using a video microscope (Olympus BX41) with DP2-BSW imaging software. The total number of nuclei in the different blood vessel layers was counted manually to determine the cellularity of the arterial wall. The pixel intensity of brown/blue staining in blood vessel sections was quantified using ImageJ software (National Institutes of Health, USA) to evaluate the amount of α-smooth muscle actin or collagen deposition, respectively.

### Structural and mechanical properties of carotid arteries

Mouse left common carotid arteries (10 mm segments upstream of the bifurcation) were mounted into a Pressure Myograph system (DMT-100P, DMT, Aarhus, Denmark) for the assessment of mechanical properties under passive conditions [53]. The blood vessels were equilibrated in calcium-free Krebs buffer solution (4.69 mM KCl, 118.99 mM NaCl, 25 mM NaHCO_3_, 5.5 mM glucose, 1.17 mM MgSO_4_, 1.18 mM KH_2_PO4 and 3 mM EDTA) kept at 37°C and aerated with 95% O_2_ and 5% CO_2_. Each segment was primed by increasing the transmural pressure stepwise (10 mmHg every three minutes) from 10 to 190 mmHg. After equilibrating at 10 mmHg for 30 minutes, transmural pressure was raised stepwise (10 mmHg increments every three minutes) to 150 mmHg. During this procedure the changes of the internal and external diameter were measured at three different locations along the arterial segment. The cross-sectional area of the arterial wall, average wall thickness and wall-to-lumen ratio were calculated using the Ion Wizard (Version 6.0, Ion Optix Corp., Milton, Massachusetts, USA) [54]. Comparisons are based on the calculations of the following parameters:

Wall cross-sectional area (CSA)

$\text{CSA}=\frac{\pi ({\text{De}}^{2}-{\text{Di}}^{2})}{4}$, where De is the external diameter and Di is the internal diameter of the vessel for a given transmural pressure.

Circumferential strain

$\varepsilon =\frac{\text{Di}-\text{Dio}}{\text{Dio}}$, where Dio represents the internal diameter at a pressure of 10 mmHg.

Circumferential stress

$\sigma =\frac{\text{P}\times \text{Di}}{2\text{WT}}$, where P is a given transmural pressure (1 mmHg = 0.133 kPa).

Elastic modulus

The incremental elastic modulus (E_inc_ or Young's modulus) describes the intrinsic elastic properties of the wall material, independently of blood vessel geometry. It was obtained by fitting the stress-strain data to the exponential curve σ = σ_o_ e^βε^, where σ_o_ is the stress at a pressure of 10 mmHg and β is a constant related to the tangent of the stress-strain curve, i.e. β is proportional to the incremental elastic modulus.

### Data calculation and statistical analysis

All calculations were performed using SPSS 19.0 software (IBM Corporation, NY, USA). All experiments were repeated at least three times. Results are presented as means ± SEM. Statistical analysis of differences was performed using Student's *t-*test for unpaired observations or one-way ANOVA with Bonferroni correction for multiple comparisons. *P* values less than 0.05 were considered to indicate statistically significant differences.

## SUPPLEMENTARY MATERIAL


