# Supplementary material for: Endothelial SIRT1 prevents adverse arterial remodeling by facilitating HERC2-mediated degradation of acetylated LKB1
Source: Oncotarget. 2016 May 29;7(26):39065–81. doi: 10.18632/oncotarget.9687 (PMC5129914; doi:10.18632/oncotarget.9687)
Supplement: Supplementary file 1 [file oncotarget-07-39065-s001.pdf]

Endothelial SIRT1 prevents adverse arterial remodeling by facilitating HERC2-mediated degradation of acetylated LKB1

Supplementary Material

Animal studies

Mice deficient in eNOS were purchased from Jackson Laboratory (Bar Harbor, ME, USA) [1]. Transgenic mice with endothelial overexpression of human SIRT1 (EC-SIRT1) were generated in-house [2]. The above two strains of mice were cross-bred for ten generations to produce mice without ($eNOS^{-/-}$) or with endothelial overexpression of human SIRT1 ($eNOS^{-/-}$EC-SIRT1). Polymerase chain reaction (PCR)-based genotyping was performed as described [3]. The animals were housed in a controlled environment (23±1 °C, 12-hour/12-hour light/dark cycle) and maintained on a standard chow diet with free access to water.

For lentiviral treatment, piLenti-HERC2siRNA-GFP ($1.6x10^{7}$ IU/mouse, Applied Biological Materials Inc., Richmond, BC, Canada) for knocking down murine *HERC2* was administrated by tail vein injection into eight-weeks old mice. The piLenti-siRNA lentivirus was used as negative control. At the end of the treatment, mice were anesthetized by intraperitoneal injection of sodium pentobarbital (100 mg/kg; H. Lundbeck A/S, Copenhagen, Denmark). Loss of consciousness and suppression of reflexes (absence of withdrawal reflex, tail-pinch response and limb muscle tone with regular heart and respiratory rates) were checked to ensure sufficient anesthesia. The carotid arteries were harvested for *in vitro* experimentation.

Antibodies and reagents

Antibodies against DYKDDDDK FLAG-tag, HA-tag, Histone H3, GAPDH, LKB1, p53 or β-actin were purchased from Cell Signaling Technology (Beverly, MA, USA). Antibody against SIRT1 was from Millipore (Billerica, MA, USA). Antibodies against HERC1 or STRADα were from Santa Cruz Biotechnology (Texas, USA). Antibodies recognizing HERC2, HERC3, TGFβ1 or α-smooth muscle actin were from Abcam (Cambridge, UK). The complete protease inhibitor cocktail tablets were from Roche (Basel, Switzerland). The E1 (UBA1), UbcH5a, FLAG-tagged ubiquitin, cycloheximide and MG132 was purchased from Sigma. Ubc13 was obtained from Boston Biochem (Cambridge, MA, USA). Mammalian expression vector encoding HA-tagged ubiquitin (HA-Ub) was obtained from Addgene (Cambridge, MA, USA).

Cell culture, transfection and subcellular fractionation

PAEC were collected from porcine aortae and cultured as described [4]. Cells between the 2nd and 3rd passages were used. MDA-MB231, HUVEC, HEK293 and PCASMC were purchased from American Type Culture Collection (Manassas, VA, USA) and maintained at 37°C in a humidified atmosphere with 5% CO_2 and 95% air. Transfection was performed with Effectene transfection reagent obtained from QIAGEN (Hilden, Germany). At the end of the treatment, cells were harvested and re-suspended in a hypotonic buffer [10 mM Tris-HCl (pH 7.5), 10 mM KCl, 1.5 mM $MgCl_2$, 0.5 mM DTT, 0.5 mM phenylmethylsulfonyl fluoride plus protease inhibitor cocktails] and incubated on ice for ten minutes. Cell membranes were disrupted by 20 passes through a 25-gauge needle and the nuclear integrity monitored under a

microscope. After centrifugation for five minutes at 1,000 *g*, the supernatant was harvested as the “cytosolic fraction”. The nuclear pellet was washed with the hypotonic buffer and lysed with RIPA lysis buffer [50 mM Tris-HCl (pH 7.4), 100 mM NaCl, 0.1% SDS, 1% Triton-X 100, 5 mM EDTA, 1 mM NaF, and 1 mM Na_3VO_4 plus protease inhibitors]. The lysates were centrifuged at 12,000 *g* for five minutes and the supernatant collected as the “nuclear fraction”. Protein concentrations were determined with the Pierce BCA Protein Assay Kit (Thermo Fisher Scientific, Waltham, MA, USA).

Cellular senescence, proliferation and apoptosis

Senescence-associated β-galactosidase (SA-β-gal) staining was performed to monitor the accumulation of senescent endothelial cells using Senescence Cells Histochemical Staining kit (Sigma, Saint Louis, MO, USA) as described [4]. Positively stained cells were counted manually from ten randomly selected areas of the images to compare the percentage of senescent cells between treatment groups.

Proliferation was evaluated either by manually counting the cells harvested at different time points after treatment using a hemocytometer under the light microscope, or by crystal violet staining as described [4]. Endothelial apoptosis was determined by the DeadEnd™ Fluorometric TUNEL system (Promega, Fitchburg, WI, USA). In brief, cells were washed with PBS, fixed with 4% formaldehyde, permeabilized with 0.2% Triton X-100, and then labeled with the reaction mixture at 37°C for 60 minutes. The nuclei of cells were visualized by staining with 4',6-

diamidino-2-phenylindole (DAPI, Life Technologies). Cells positively stained with the TUNEL reagent were counted under the fluorescence microscope.

RNA extraction and quantitative real-time PCR (QPCR)

Total RNA was isolated using Trizol Reagent (Life Technologies) according to the manufacturer's instructions. The purity and concentration of total RNA were measured with a spectrophotometer at 260 nm and 280 nm. Ratios of absorption (260/280 nm) of all samples were between 1.8 and 2.0. A QuantiTect Reverse Transcription kit from QIAGEN was used to produce cDNA. Quantitation of target genes was performed using SYBR Green PCR Master Mix (QIAGEN) and a StepOnePlus™ Real-Time PCR Systems (Applied Biosystems, Foster City, CA, USA). Primers are listed in Supplementary Table 2. Quantification was achieved using Ct values that were normalized with β-actin or GAPDH as a reference control.

Chromatin immunoprecipitation quantitative PCR (ChIP-qPCR)

HUVEC cells ($2x10^6$) under different treatment conditions were cross-linked with 1% formaldehyde at room temperature for ten minutes with shaking. After adding glycine to a final concentration of 125 mM, cells were collected for sonication to obtain the chromatin fragments of 500-1000 bp. The lysates were then precleared with salmon sperm DNA and protein A agarose beads before incubating without or with specific antibodies at 4 ℃ overnight with rotation. The chromatin fragments were eluted into a buffer containing 1% SDS and 0.1 M $NaHCO_3$. After reversed crosslinking and incubation with RNase A/proteinase K, DNA was purified by

phenol/chloroform extraction and then resuspended for ChIP-qPCR analysis. QPCR were performed using 10% of the DNA samples and quantified by using the Ct values for normalization against the input control, for which 1% of the DNA samples were used for QPCR. The resulting products were also analyzed by agarose gel electrophoresis and densitometry (MultiAnalyst Software, Bio-Rad). Primers were designed by referring to the DNA sequences of the four regulatory regions of the *TGFβ1* promoter [5], and are listed in Supplementary Table 3.

Recombinant protein purification

The prokaryotic vectors were transformed into BL21 competent cells (Life Technologies). The expression of His- or GST-tagged recombinant proteins was induced by the addition of 1 mM of isopropyl-β-D-thiogalactopyranoside (IPTG, GE Healthcare, Little Chalfont, UK). His-tagged SIRT1 or LKB1 were purified from bacterial lysates using Ni-NTA agarose beads (QIAGEN) according to the manufacturer's instructions. GST-tagged HERC2-NH_2 and HERC2-CPH proteins were purified using Glutathione Sepharose™ 4B beads (GE Healthcare) with the elution buffer containing 10 mM reduced glutathione in 50 mM Tris-HCl (pH8.0). Desalting and buffer exchange were performed using Amicon Ultra-2 mL Centrifugal Filters purchased from Millipore (Billerica, Massachusetts, USA). *In vitro* pull-down experiment was performed to evaluate protein-protein interactions. Briefly, agarose beads bound with GST-tagged proteins were incubated with cell lysates containing 500 μg proteins at room temperature for two hours under agitation. After washing

extensively with cold phosphate-buffered saline (PBS), the interacting proteins were eluted directly into SDS-PAGE loading buffer for subsequent analysis.

Immunoprecipitation and *in vitro* ubiquitination assay

Cells or subcellular fractions were solubilized in RIPA buffer [50 mM Tris-HCl (pH 7.4), 100 mM NaCl, 0.1% SDS, 1% Triton-X 100, 5 mM EDTA, 1 mM NaF, and 1mM Na_3VO_4 plus protease inhibitors] and sonicated on ice. The lysates containing 500 μg proteins were used for co-immunoprecipitation. In brief, after pre-clearing with protein A agarose beads slurry (Thermo Fisher Scientific), the supernatant was incubated overnight with the primary antibodies (2.5 μg) at 4°C under gentle agitation. Samples were subsequently mixed with 100 μl of 50% protein A agarose beads slurry and incubated at room temperature under rotary agitation for two hours. After washing three times with cold PBS, the beads were collected for *in vitro* ubiquitination assay or eluted with SDS-PAGE loading buffer for Western blotting. For evaluating the status of ubiquitination on individual proteins, RIPA buffer containing 1% SDS was used for cell lysis under denaturing conditions and then diluted to the same buffer containing 0.2% SDS for subsequent immunoprecipitation.

Western blotting

The procedures were carried out as described [2, 6]. In brief, equal amounts of proteins were separated by SDS-PAGE, transferred to a PVDF membrane and probed with specific antibodies to determine the expression of the target proteins. Band intensities were measured and quantified by densitometry using a GS-700 Imaging Densitometer (Bio-Rad, Hercules, CA, USA). Quantification of Western blots was

based on the ratio of target protein to β-actin, histone H3 or GAPDH for samples derived from total cell lysates, nuclear and cytosolic fractions, respectively.

Immunofluorescence analysis

Cells cultured on coverslips were fixed with cold methanol/acetone (1:1 vol/vol) for 15 minutes and then permeabilized with 1% Triton X-100 in PBS for five minutes. After blocking with 3% BSA for one hour, cells were incubated overnight with specific primary antibodies at 4°C. After washing with PBS, cells were then incubated with Alexa Fluor 594–labeled secondary antibodies (Life Technologies) at dilutions of 1:500 for one hour at room temperature in the dark. The slides were mounted with ProLong Gold antifade reagent with DAPI (Life Technologies). Fluorescent images were taken with a BX-41 fluorescent microscope (Olympus, Tokyo, Japan).

Arterial blood pressure measurement

Arterial blood pressure was determined using a computerized tail-cuff BP-2000 Blood Pressure Analysis System (Visitech Systems Inc, Apex, NC, USA). All recordings were obtained between 15:00 and 17:00, after the animals had been warmed in a 35°C chamber for ten minutes. The mice were habituated to this procedure for three days before the actual experiment. The systolic and diastolic blood pressures were recorded and averaged from at least ten consecutive readings.

***In vitro* de-acetylation assay**

Acetylated [LIGK(Ac)YLMGDLLGEGSYGKVKEV (AcLys48) and LIGKYLMGDLLGEGSYGKVK(Ac)EV (AcLys64)] and non-acetylated

[LIGKYLMGDLLGEGSYGKVKEV] LKB1 peptides were synthesized by GenScript USA Inc. (Piscataway, NJ, USA) and used for *in vitro* de-acetylation assay as described [3]. In brief, recombinant human SIRT1 (3.6 μM) was incubated with peptide (500 μM) and NAD^+ (500 μM) in 50 mM Tris-HCl (pH 7.5) for one hour at 37°C. The reaction mixtures were analyzed by a reversed-phase HPLC on a Symmetry® C18 column (5 µm, 3.9 x 150 mm, Waters). The mobile phase consisted of solvent A (0.5% trifluoroacetic acid) and B (acetonitrile with 0.5% trifluoroacetic acid). A 40-minute, 0-40% linear gradient was used for the separation at a flow rate of 1 ml/min. Chromatographic peaks were monitored at 214 nm by a UV detector.

Pulse-Wave Doppler measurement

Mice were anesthetized by intraperitoneal injection of 100 mg/kg Ketamine and 0.2 mg/kg Xylazine (Alfasan International, Woerden, The Netherland). Blood flow was measured in the right common carotid artery using the Vevo 2100 system (Visual Sonics, Toronto, ON, Canada). Peak systolic velocities (PSVs) and end-diastolic velocities (EDVs) were measured in pulsed-wave Doppler mode at 30 MHz 2-3 mm before carotid bifurcation. The resistive index (RI) was calculated as RI=(PSV-EDV)/PSV [7].

References

1. Shesely EG, Maeda N, Kim HS, Desai KM, Krege JH, Laubach VE, Sherman PA, Sessa WC and Smithies O. Elevated blood pressures in mice lacking endothelial

nitric oxide synthase. Proceedings of the National Academy of Sciences of the United States of America. 1996; 93(23):13176-13181.

2. Zu Y, Liu L, Lee MY, Xu C, Liang Y, Man RY, Vanhoutte PM and Wang Y. SIRT1 promotes proliferation and prevents senescence through targeting LKB1 in primary porcine aortic endothelial cells. Circulation research. 2010; 106(8):1384-1393.

3. Xu C, Cai Y, Fan P, Bai B, Chen J, Deng HB, Che CM, Xu A, Vanhoutte PM and Wang Y. Calorie restriction prevents metabolic aging caused by abnormal SIRT1 function in adipose tissues. Diabetes. 2015; 64(5):1576-1590.

4. Bai B and Wang Y. Methods to investigate the role of SIRT1 in endothelial senescence. Methods in molecular biology. 2013; 965:327-339.

5. Kim SJ, Glick A, Sporn MB and Roberts AB. Characterization of the promoter region of the human transforming growth factor-beta 1 gene. The Journal of biological chemistry. 1989; 264(1):402-408.

6. Bai B, Liang Y, Xu C, Lee MY, Xu A, Wu D, Vanhoutte PM and Wang Y. Cyclin-dependent kinase 5-mediated hyperphosphorylation of sirtuin-1 contributes to the development of endothelial senescence and atherosclerosis. Circulation. 2012; 126(6):729-740.

7. Anea CB, Ali MI, Osmond JM, Sullivan JC, Stepp DW, Merloiu AM and Rudic RD. Matrix metalloproteinase 2 and 9 dysfunction underlie vascular stiffness in circadian clock mutant mice. Arteriosclerosis, thrombosis, and vascular biology. 2010; 30(12):2535-2543.

Supplementary Table 1. Sequences of primers used for cloning the HERC2 sub-domains.

Domain names	Expression vectors	Primer sequence	Product size
**HERC2-DOC**	DOC-3FLAG	Forward 5’- ATAAGAATGCGGCCGCATGCAGCTGAAGCGTTGCCACAG-3’	**0.5kb**
		Reverse 5’- CCGCTCGAGCAGCAGGATGAGACCATGGATTTT-3’	
**HERC2-RCC2**	RCC2-3FLAG	Forward 5’-ATAAGAATGCGGCCGCATGAAGAGCCTGAATGTGTCG-3’	**2.5kb**
		Reverse 5’-CCGCTCGAGAAATTCTACCCTGAGTCCTT-3’	
**HERC2-HECT**	HECT-3FLAG	Forward 5’-ATAAGAATGCGGCCGCATGCTGAACCGCATCCAGG-3’	**1.2kb**
		Reverse 5’-CCGCTCGAGGTGTCCTGTTAAATAATCTTG-3’	

Supplementary Table 2. Sequences of primers used for QPCR analysis.

Gene name	Gene symbol	Species	Accession ID	Primer sequences
GAPDH	*Gapdh*	*Mus musculus*	GI:576080553	Forward 5'- AACTTTGGCATTGTGGAAGGG - 3'
				Reverse 5'- GGATGCAGGGATGATGTTCT -3'
MMP9	*Mmp9*	*Mus musculus*	GI:530719523	Forward 5'-CAAAGACCTGAAAACCTCCAAC-3'
				Reverse 5'-GACTGCTTCTCTCCCATCATCT-3'
TGFβ1	*Tgfb1*	*Mus musculus*	GI:6755774	Forward 5'- CACCATCCATGACATGAACC- 3'
				Reverse 5'- ACTTCCAACCCAGGTCCT TC- 3'
Collagen I	*Col1a1*	*Mus musculus*	GI:118131144	Forward 5'-ATCTCCTGGTGCTGATGGAC- 3'
				Reverse5'-ACCTTGTTTGCCAGGTTCAC- 3'
Collagen III	*Col3a1*	*Mus musculus*	GI:226423932	Forward 5'-AGGCAACAGTGGTTCTCCTG- 3'
				Reverse 5'-GACCTCGTGCTCCAGTTAGC- 3'
HERC1	*Herc1*	*Mus musculus*	GI:257196143	Forward 5'-TGACAGCTGGCAAAATCTTTCA- 3'
				Reverse 5'-CTGCTGAGACTGTGGAGGTG- 3'
HERC2	*Herc2*	*Mus musculus*	GI:134288897	Forward 5'-GAGCTGGGCCGAAGGAG- 3'
				Reverse 5'-CTCTTGTGAATGCAAGCTGGA- 3'
HERC3	*Herc3*	*Mus musculus*	GI:188035868	Forward 5'-GCAGTGCAGAGAAAAAGGCTAT- 3'
				Reverse 5'-GGAACACGGAGTGGTTTCCT - 3'
HERC4	*Herc4*	*Mus musculus*	GI:313103014	Forward 5'-GACGGCTTGTCTCTCGGTC- 3'
				Reverse 5'-GTTCTTATCGGGTCTCTCAGGG- 3'
HERC6	*Herc6*	*Mus musculus*	GI:253314513	Forward 5'-AGGAAAGGTCTTCGCTTGGG- 3'
				Reverse 5'-AACACGTGGCCATCTTCTGA- 3'
β-actin	*ACTB*	*Sus scrofa*	GI:545892687	Forward 5'- CTGGGGCCTAACGTTCTCAC -3'
				Reverse 5'- GTCCTTTCTTCCCCGATGTT -3'
VCAM1	*VCAM1*	*Sus scrofa*	GI:47522703	Forward 5'-CCACGCTGGTCATGAATCCCGT-3'
				Reverse 5'-AACGTCAGGCACCAGACACCTGA-3'
SELE	*SELE*	*Sus scrofa*	GI:47523601	Forward 5'-GCTCGCCTTGTGCTACACAGCT-3'
				Reverse 5'-GAGGCTTTGGGGACATGTCACG-3'
TGFβ1	*TGFB1*	*Sus scrofa*	GI:55741796	Forward 5'-CGCATCGAGGCCATTCGCGGCCAGATTC-3'
				Reverse 5'-TCAGCCACTGCCGCACAACTCCGGTGAC-3'

MMP9	*MMP9*	*Sus scrofa*	GI:115490796	Forward 5'-CCACAGGCCCTCCTTCAG-3'
				Reverse 5'-TGAACAGCAGCACCTTACC-3'
HERC1	*HERC1*	*Sus scrofa*	GI:927095680	Forward 5'- TGAGGAGAGTTTCCATGAGATGA -3'
				Reverse 5'- ACAGGGACGATCCAGGACAT -3'
HERC2	*HERC2*	*Sus scrofa*	GI: 927209854	Forward 5'-ATGTTCATGGAACGCCTGGT-3'
				Reverse 5'-ACAACATTTGGCTCAGCGTG-3'
HERC3	*HERC3*	*Sus scrofa*	GI:545846738	Forward 5'-CTAACCTGCAGGGGATCGTG-3'
				Reverse 5'-TGCTCCAATTTGCTCTGGCT -3'
HERC4	*HERC4*	*Sus scrofa*	GI:545870349	Forward 5'-TTCTCTGAGGAGCCCGGTAA-3'
				Reverse 5'-TATCCCTATGTCCGGCCCC-3'
HERC6	*HERC6*	*Sus scrofa*	GI:545893179	Forward 5'-TGGTGCTTGCCAGTCAGTAT-3'
				Reverse 5'-CCCCTCTAATTCCGTCGCTG-3'

Supplementary Table 3. Sequences of primers used for ChIP-PCR analysis of *TGFβ1* promoter.

Name	Region (from transcriptional start site)	Species	Gene ID	Primer sequences	Product Size
Negative regulatory region 1 (NRR1)	*-1362 to -1132*	*Homo sapiens*	7040	Forward 5'-ATCACTTTGGCTGCTGTGTG-3'<br>Reverse 5'-GGCCCAGTCTTTTCCTCTCT-3'	104bp
Negative regulatory region 2 (NRR2)	*-731 to -453*	*Homo sapiens*	7040	Forward 5'-GGCCCAGTTTCCCTATCTGT-3'<br>Reverse 5'-CCTTTACTGAGCACCTCCCA-3'	104bp
Positive regulatory region1 (PRR1)	*-453 to -323*	*Homo sapiens*	7040	Forward 5'-GTTTCCCAGCCTGACTCTCC-3'<br>Reverse 5'-ACCAGAGAAAGAGGACCAGG-3'	108bp
Positive regulatory region 2 (PRR2)	*+1 to+271*	*Homo sapiens*	7040	Forward 5'-ATCTCCCTCCCACCTCCC-3'<br>Reverse 5'-CGGCTCGTCTCAGACTCTG-3'	182bp

Supplementary Figure 1

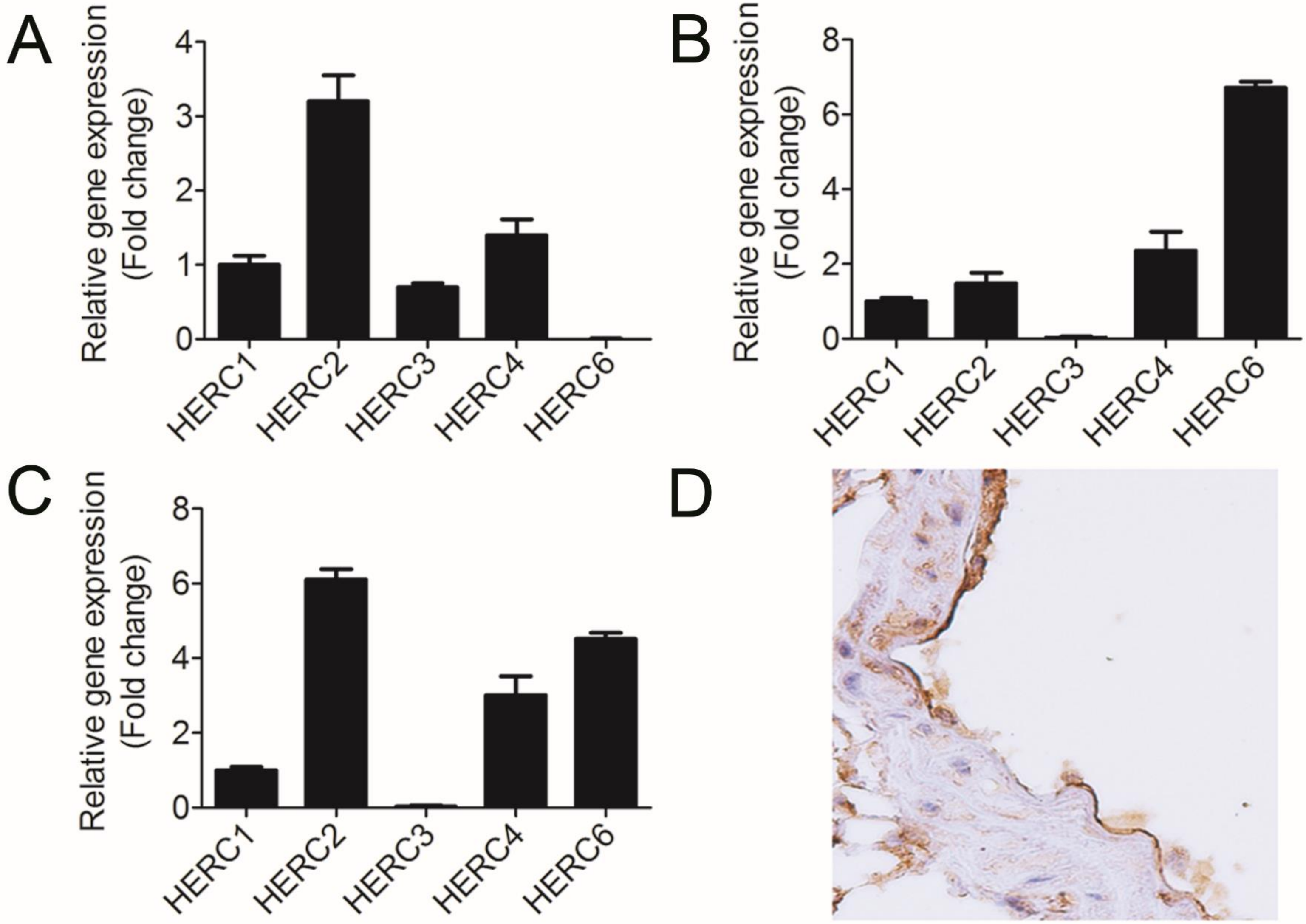

Supplementary Figure 1. A, QPCR was performed in PAEC for measuring the relative gene expression levels of porcine *HERC* family members, including *HERC1*, *HERC2*, *HERC3*, *HERC4* and *HERC6*. **B,** QPCR was performed in carotid arteries of wild type mice for comparing the gene expression levels of the murine *HERC* family members. **C,** QPCR was performed in samples derived from the endothelial layer of carotid arteries of wild type mice, which were flushed out by TRIZOL reagent, for comparing the gene expression levels of the *HERC* family members. All QPCR results are presented as fold changes versus *HERC1* gene expression. **D,** Immunohistochemistry was performed for evaluating the protein expression pattern of HERC2 in mice carotid arteries.

Supplementary Figure 2

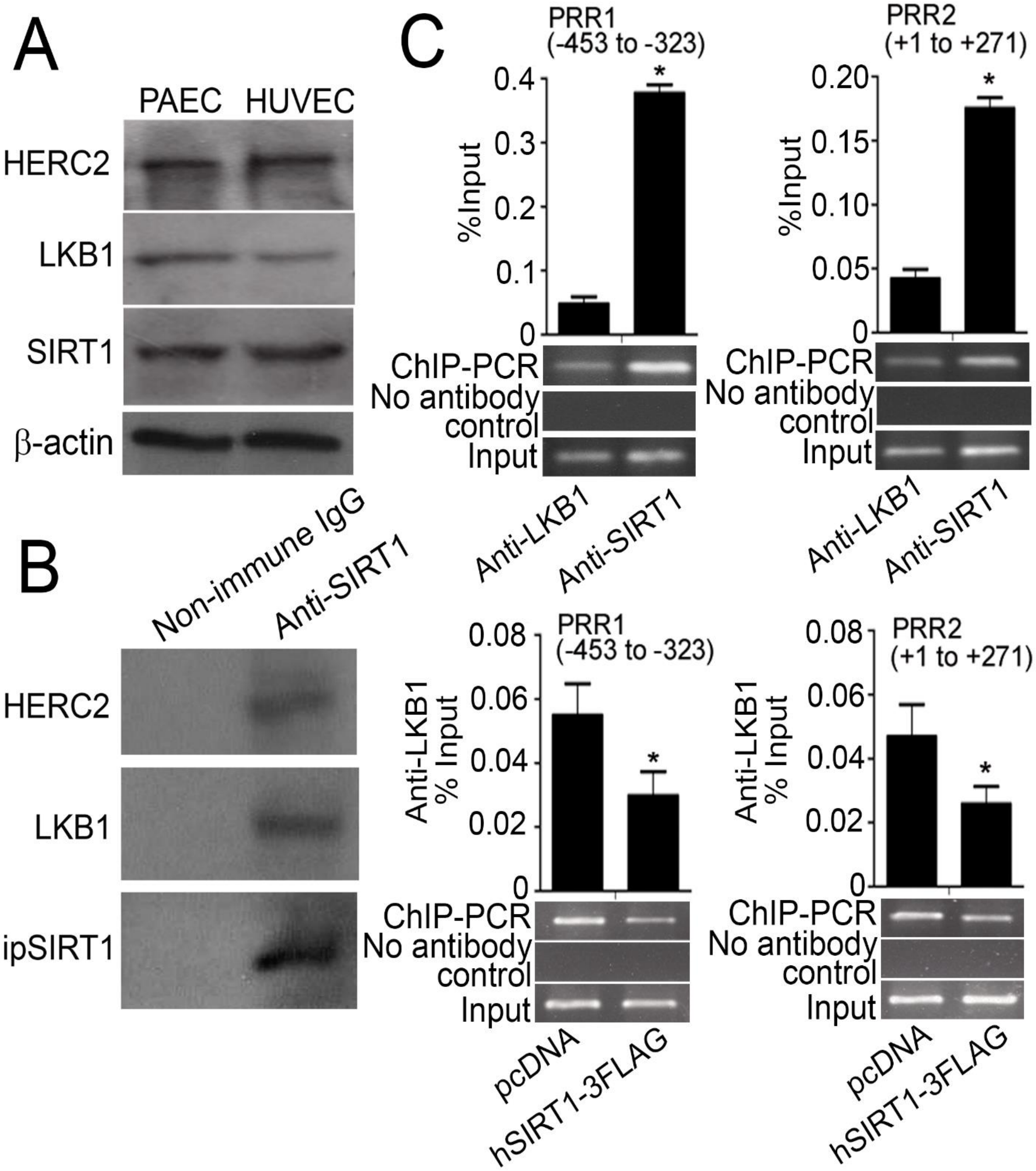

Supplementary Figure 2. A, Western blotting was performed to detect the protein expressions of HERC2, LKB1 or SIRT1 in cell lysates collected from PAEC and HUVEC cultures. **B,** Co-immunoprecipitation was performed in HUVEC lysates using non-immune IgG or anti-SIRT1 antibodies. The absence or presence of HERC2 and LKB1 in the precipitated immunocomplexes was confirmed by Western blotting. **C,** ChIP-qPCR was performed to evaluate the associations of LKB1 or SIRT1 protein with the PRR1 and PRR2 regions of *TGFβ1* promoter, respectively, in non-transfected HUVEC (top panels). After transient transfection with pcDNA or hSIRT1-3FLAG, ChIP-qPCR was performed in HUVEC to evaluate the association of LKB1 with PRR1 or PRR2 (bottom panels). Bar charts represent the quantitative comparison of Ct values of each reaction after normalization against the input DNA samples.

Supplementary Figure 3

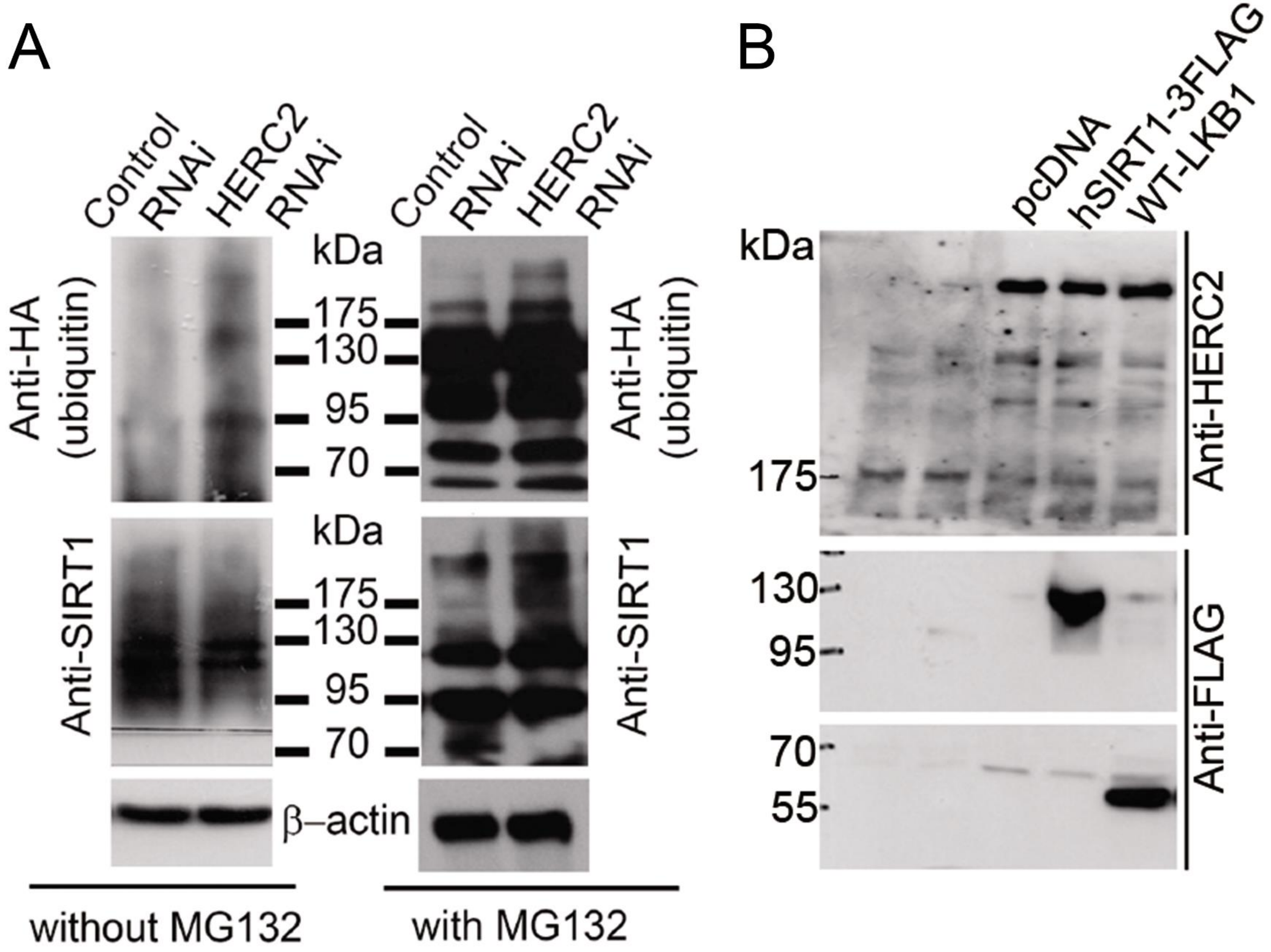

Supplementary Figure 3. A, PAEC were transfected with HA-Ub together with control RNAi or HERC2 RNAi for 72 hours, and treated or not with MG132 (10 μM) during the last six hours. Cells were subjected or not to immunoprecipitation and then lysed for SDS-PAGE and Western blotting to measure the ubiquitinated (top panels) and total (bottom panels) SIRT1 using anti-HA-tag and anti-SIRT1 antibodies, respectively. β-actin was probed as the loading control for total cell lysates. **B,** The protein expression of HERC2 was measured by Western blotting in PAEC transiently transfected with pcDNA, hSIRT1-3FLAG, or LKB1-WT-3FLAG. Overexpression of FLAG-tagged SIRT1 or LKB1 was confirmed by probing with anti-FLAG antibodies.

Supplementary Figure 4

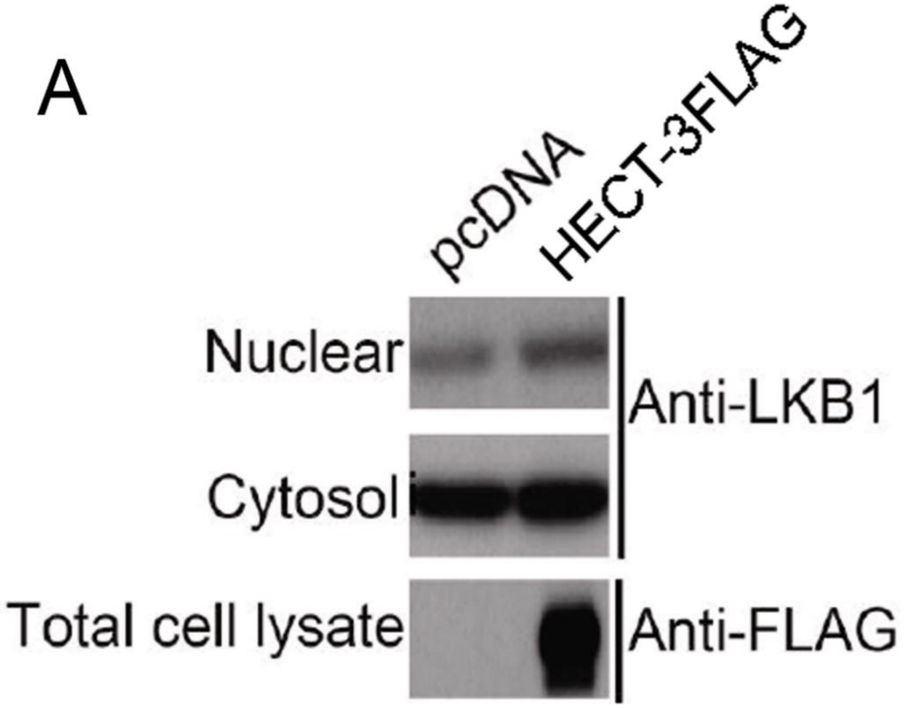

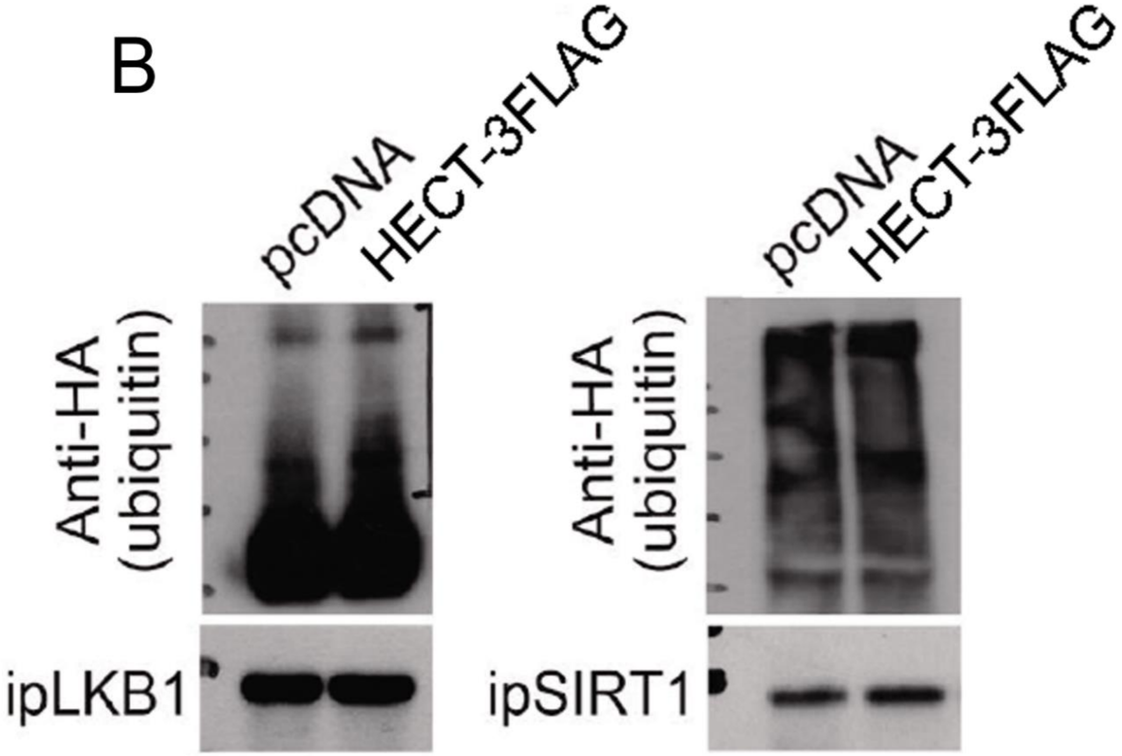

Supplementary Figure 4. The pcDNA or HECT-3FLAG vectors were used for transient transfections in PAEC. **A**, Forty-eight hours after transfection, nuclear and cytosolic fractions were collected to detect the amount of LKB1 by Western blotting. Overexpression of the HERC2-HECT domain was confirmed in total cell lysates, by Western blotting using an anti-FLAG antibody. **B**, HA-Ub was co-transfected in the cells of panel A. After treatment with MG132 (10 μM, six hours), cells were lysed under denaturing conditions for co-immunoprecipitation using antibodies against LKB1 or SIRT1. The presence of HA-tagged polyubiquitin was determined in the immunocomplexes by Western blotting using anti-HA-tag antibody.

Supplementary Figure 5

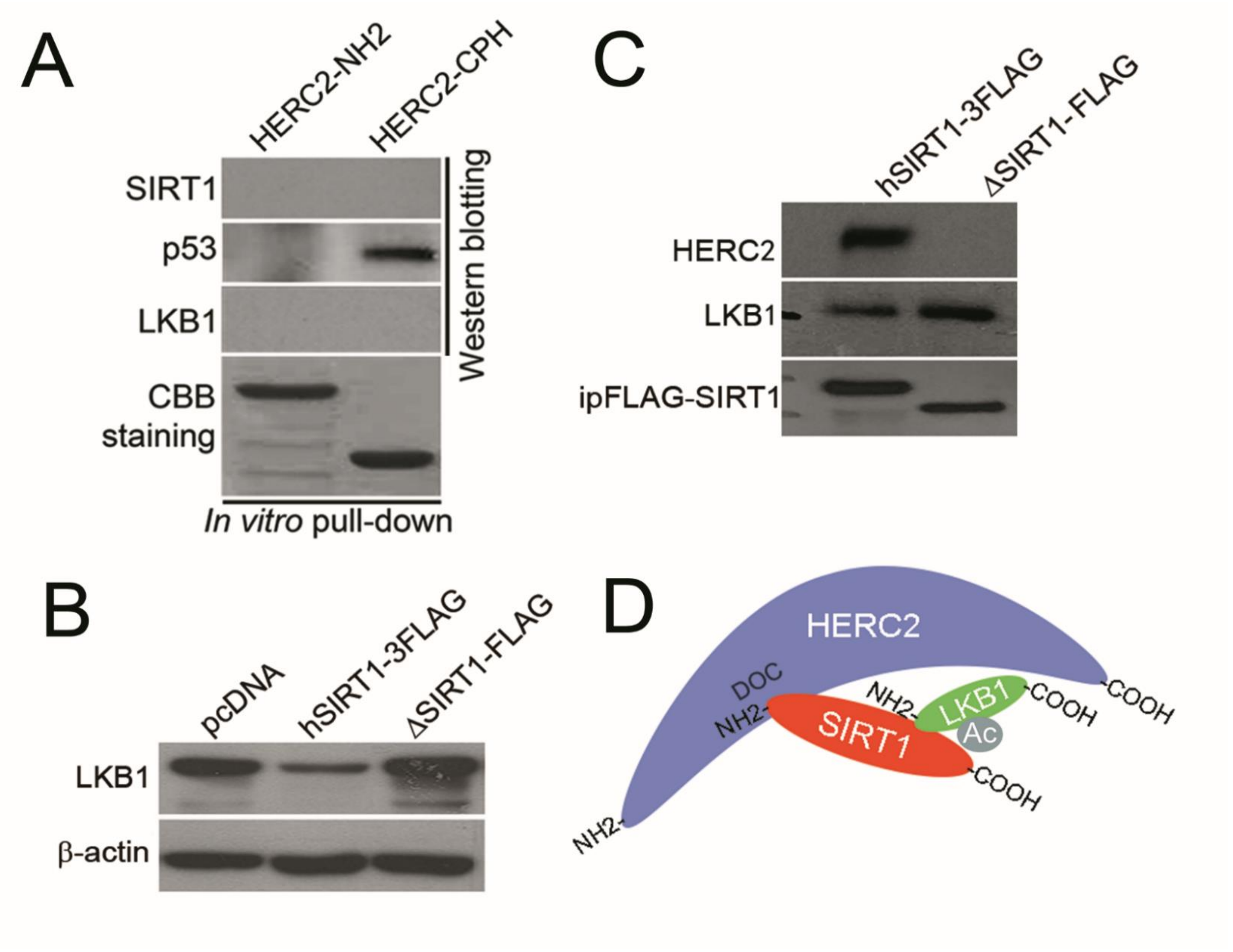

Supplementary Figure 5. A, *In vitro* pull-down experiments were performed by incubating the whole cell lysates of PAEC with purified GST-tagged HERC2-NH_2 or HERC2-CPH domains. Western blotting was performed to detect the associations of SIRT1, LKB1 or p53 with the GST-tagged HERC2 subdomains. **B,** The pcDNA, hSIRT1-3FLAG or ΔSIRT1-FLAG vectors were used for transient transfections in PAEC. At 48 hours after transfection, cells were collected for Western blotting to measure the total protein contents of LKB1. β-actin was probed as the loading control. **C,** Co-immunoprecipitation was performed in PAEC overexpressing FLAG-tagged full-length or truncated human SIRT1 prepared as in panel B, using an antibody recognizing the FLAG tag. The presence of HERC2 and LKB1 in the precipitated immunocomplexes was determined by Western blotting using specific antibodies. **D,** A schematic illustration of the interaction stoichiometry of HERC2/SIRT1/LKB1 complexes, which are formed between different domains/regions of the three molecules.

Supplementary Figure 6

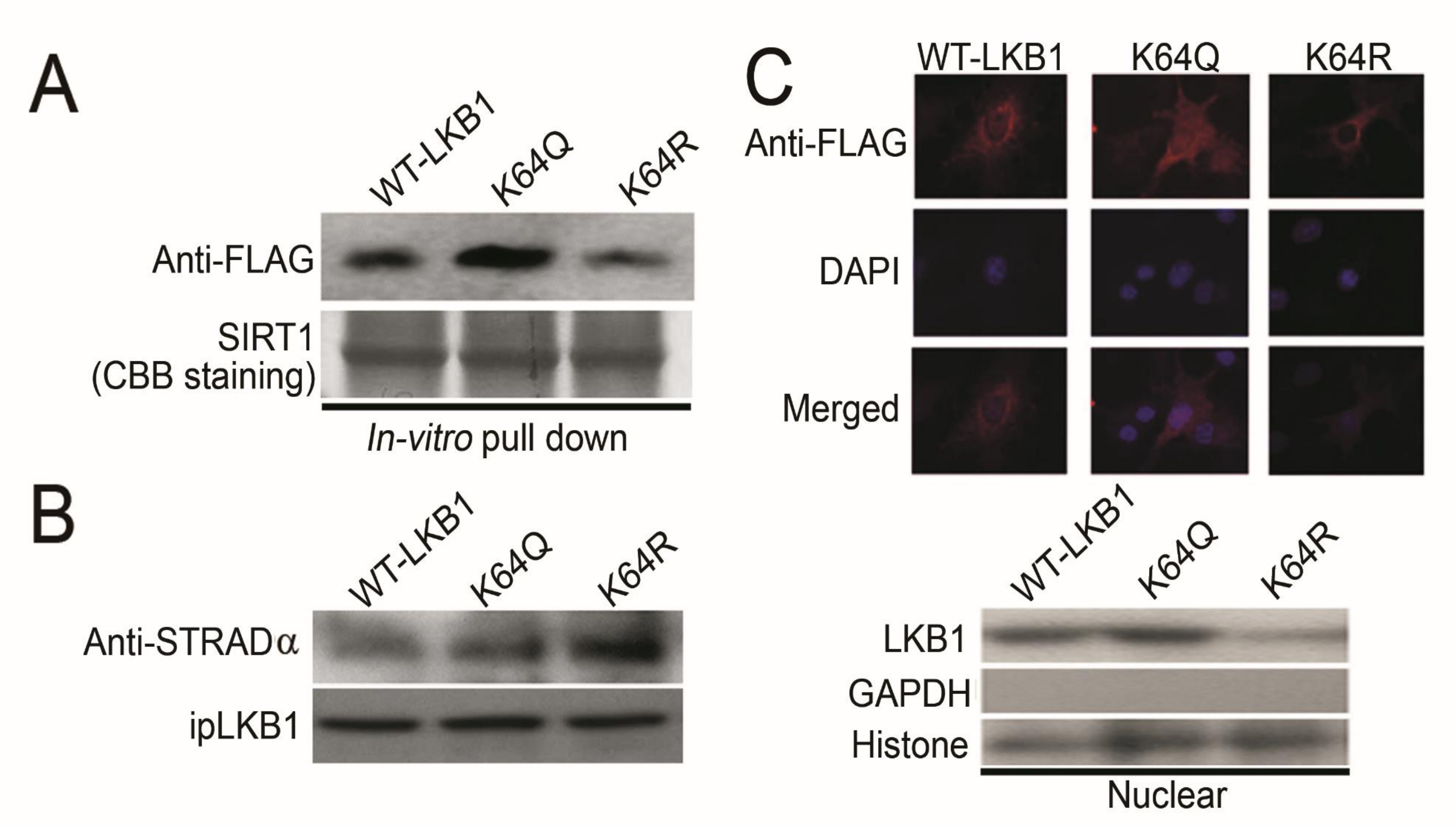

Supplementary Figure 6. **A,** PAEC were transfected with vectors encoding WT-LKB1, K64Q or K64R. Forty-eight hours after transfection, *in vitro* pull-down experiments were performed by incubating the whole cell lysates with purified GST-tagged SIRT1 proteins. Western blotting was performed to detect the presence of different LKB1 variants in the eluted samples using an anti-FLAG antibody. **B,** PAEC were transfected as in panel A. Forty-eight hours after transfection, cell lysates were collected to perform co-immunoprecipitation using an antibody recognizing LKB1. The presence of STRADα in the immunocomplexes was determined by Western blotting using specific antibodies. **C,** PAEC were transfected as in panel A. Forty-eight hours after transfection, the intracellular localizations of WT-LKB1, K64Q or K64R were examined by immunofluorescence staining using anti-FLAG antibodies (Top panel; Nuclei were stained with DAPI. Magnification, 400x). Western blotting was also performed to detect the protein presence of WT-LKB1, K64Q and K64R in the nuclear fractions of the transfected cells.

Supplementary Figure 7

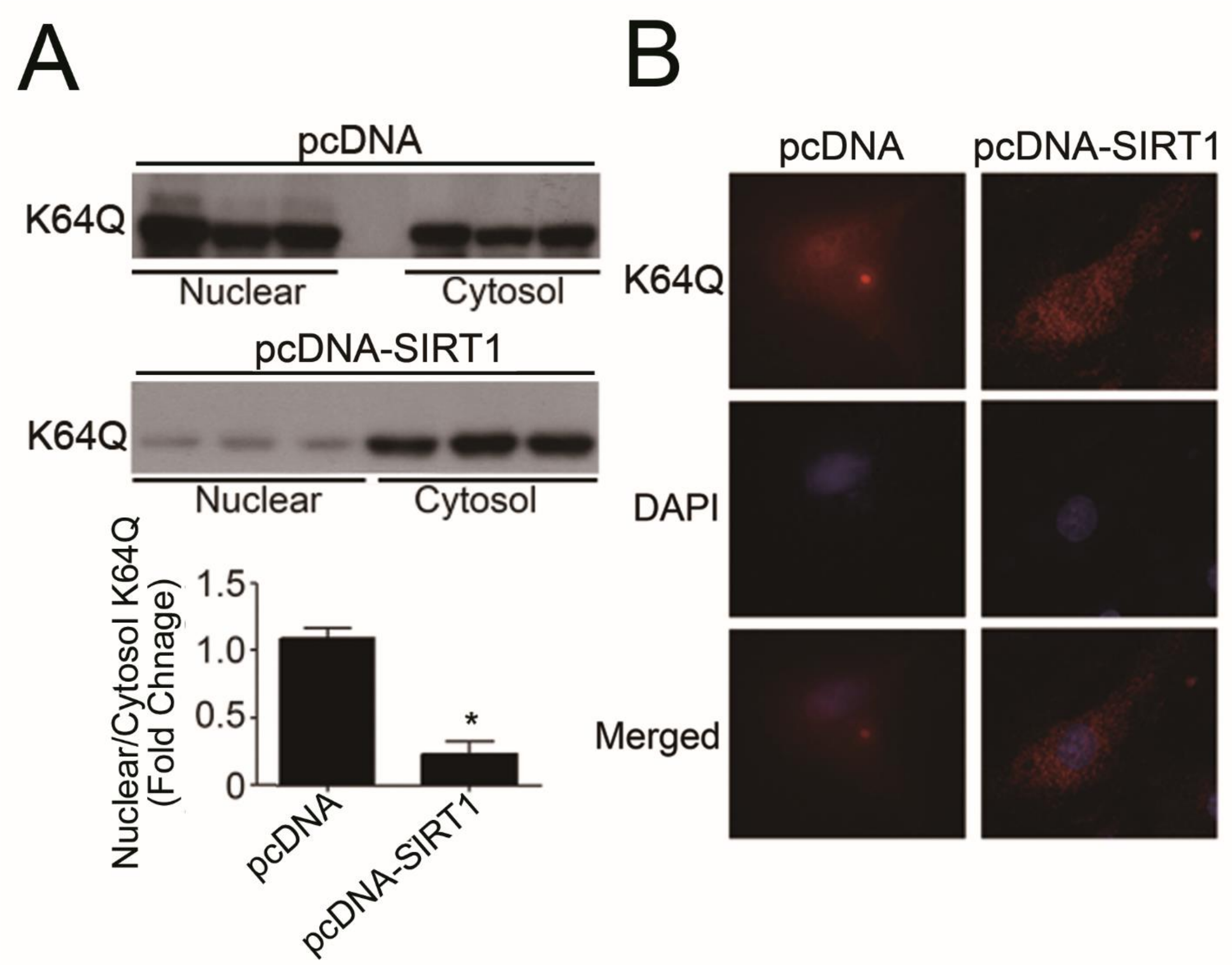

Supplementary Figure 7. A, Transient transfection was performed in PAEC to overexpress K64Q, or K64Q together with non-tagged SIRT1. The nuclear and cytosolic fractions were collected and subjected to Western blotting to measure the amount of FLAG-tagged LKB1-K64Q using an anti-FLAG antibody. The ratio of K64Q in nuclear and cytosolic fractions was calculated for comparison between cells without and with SIRT1 overexpression. *, $P<0.05$ cells overexpressing SIRT1 and K64Q vs cells overexpressing only K64Q (n=3). **B,** PAEC were transfected with vectors encoding K64Q or K64Q together with non-tagged SIRT1 to evaluate the intracellular localizations of LKB1 by immunofluorescence using an anti-FLAG antibody. Nuclei were stained by DAPI. Magnification, 400x.

Supplementary Figure 8

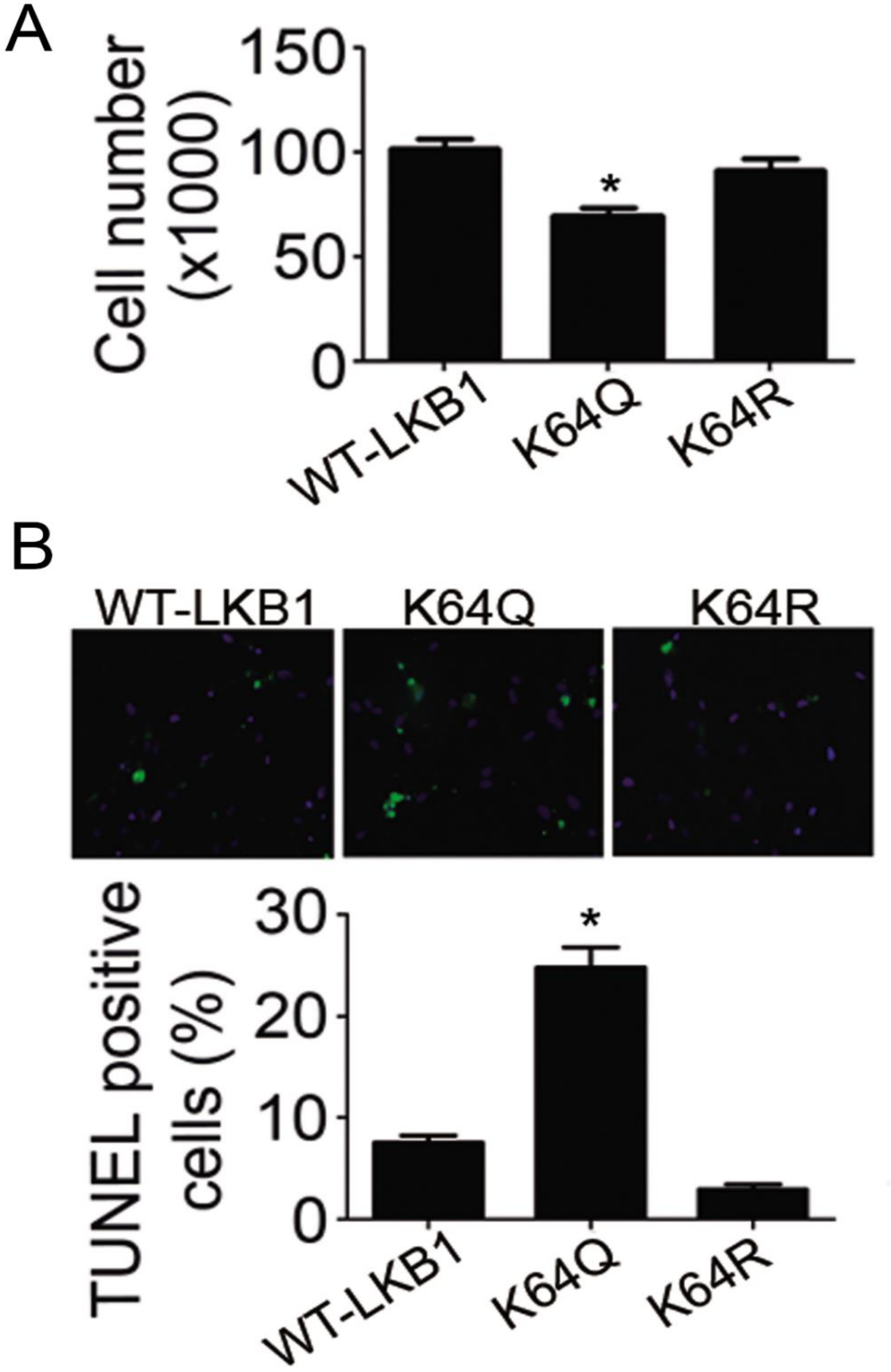

Supplementary Figure 8. Proliferation and apoptosis were evaluated in PAEC overexpressing WT-LKB1, K64Q or K64R. **A,** Cells were seeded in 24-well plates for transient transfections with vectors encoding WT-LKB1, K64Q or K64R. After 48 hours of culture, the number of cells in each well was counted manually and presented as percentage changes for comparison. **B,** The number of apoptotic cells was determined by TUNEL staining in cultures after overexpressing WT-LKB1, K64Q or K64R for 48 hours. The positively stained cells were counted manually and calculated as percentage of the total cell number. *, $P<0.05$ vs WT-LKB1 group (n=4).

Supplementary Figure 9

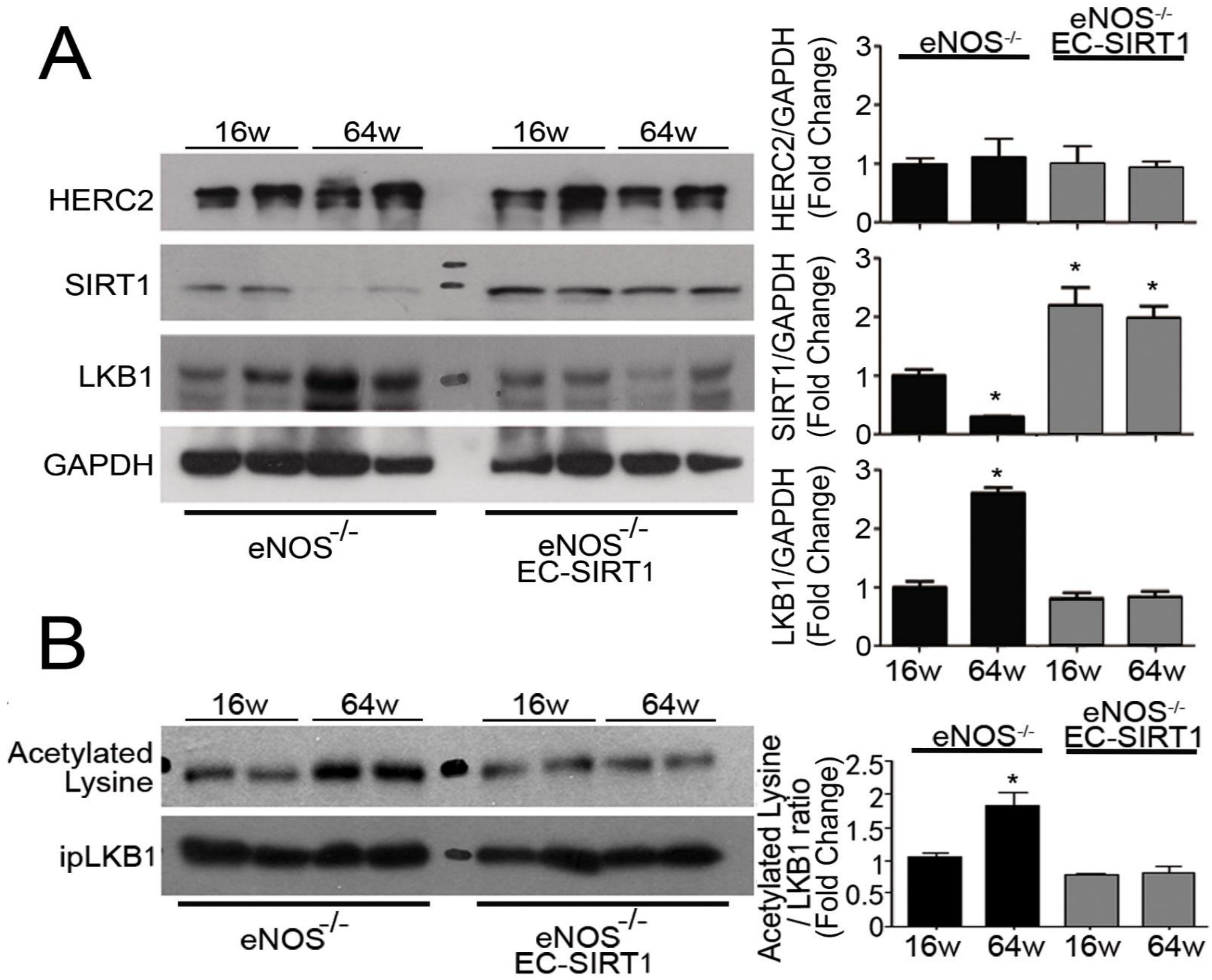

Supplementary Figure 9. A, Carotid arteries from 16- and 64-weeks old eNOS$^{-/-}$ and eNOS$^{-/-}$EC-SIRT1 mice were collected and subjected to Western blotting to examine the protein levels of HERC2, SIRT1 and LKB1. **B,** Arteries collected as in Panel A were lysed for co-immunoprecipitation using an antibody recognizing LKB1. Western blotting was performed to detect the amount of acetylated-LKB1 using an antibody recognizing acetylated-lysine residues. The ratios between the amounts of acetylated and total LKB1 in the immunoprecipitates were calculated for comparison. *, $P<0.05$ vs 16-weeks old eNOS$^{-/-}$ mice (n=3).

Supplementary Figure 10

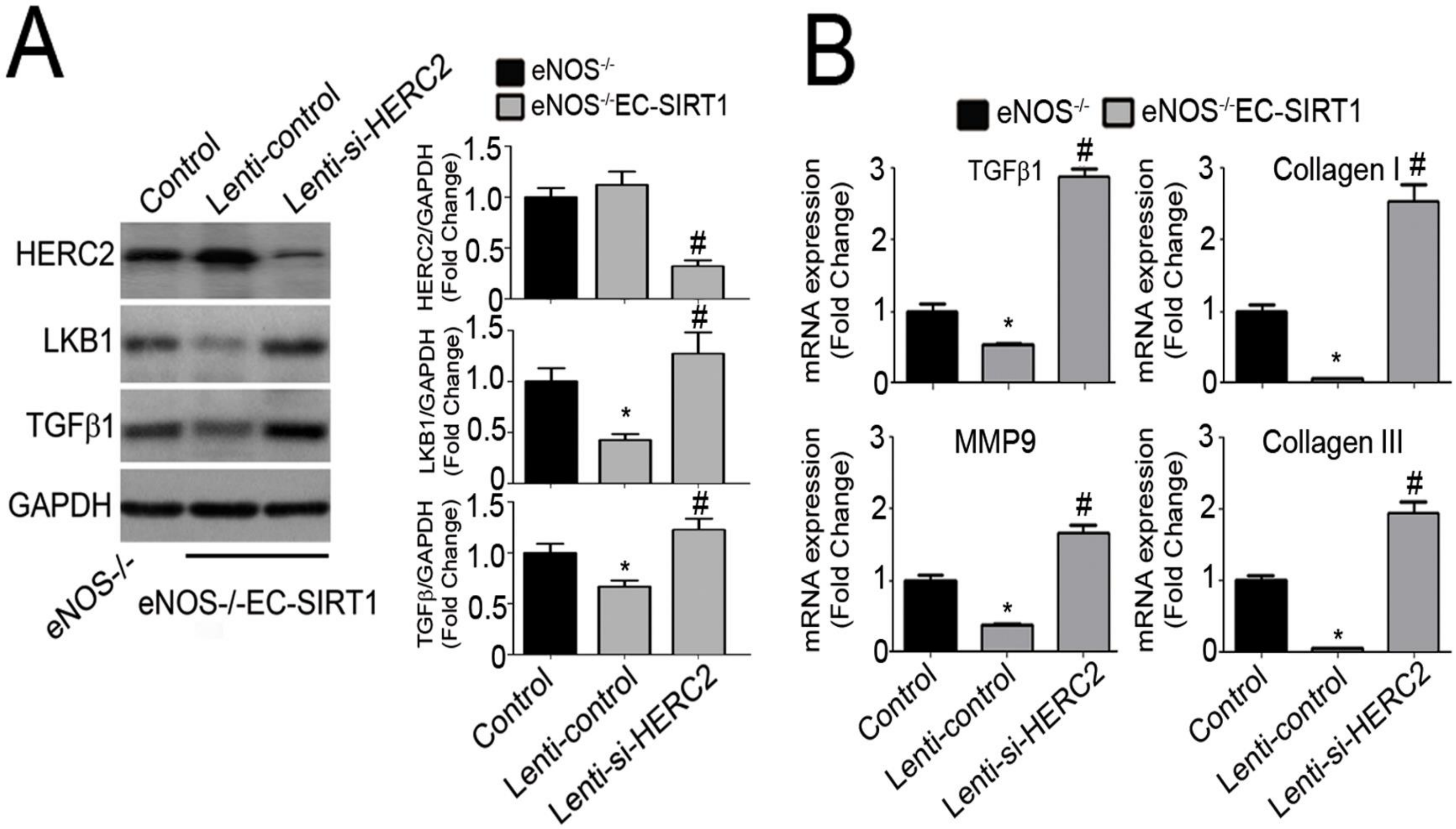

Supplementary Figure 10. A, After lentiviral treatment for 24 weeks, the carotid arteries collected from eNOS$^{-/-}$ [treated with Lenti-control] and eNOS$^{-/-}$EC-SIRT1 [treated with Lenti-control or Lenti-si-HERC2] mice were subjected to Western blotting to measure the protein levels of HERC2, LKB1 and TGFβ1. **B,** RNA was extracted from the carotid arteries collected as in panel A. QPCR was performed for analyzing the mRNA expressions of genes involved in arterial remodeling. *, P<0.05 vs control (eNOS$^{-/-}$ mice); #, P < 0.05 vs Lenti-control-treated eNOS$^{-/-}$EC-SIRT1 mice (n=5-6).

Supplementary Figure 11

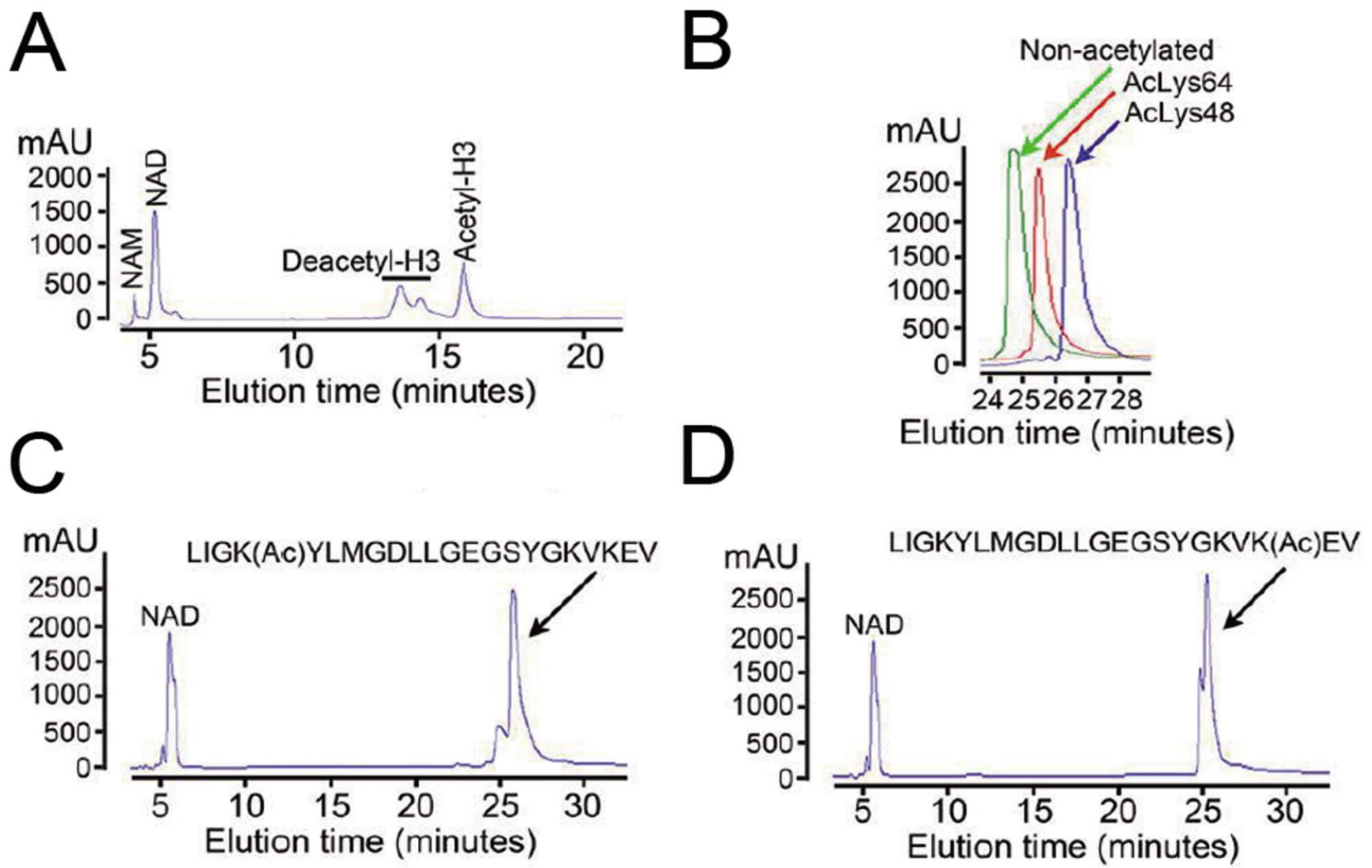

Supplementary Figure 11. The deacetylase activity of SIRT1 was evaluated using an *in vitro* assay followed by reversed-phase HPLC analysis. **A**, Recombinant human SIRT1 was incubated with acetylated H3 peptide [ARTKQTARKSTGGK(Ac)APPKQLC] and NAD+ for evaluating the production of deacetylated H3 peptide as described [3]. **B,** The three LKB1 peptides, including the non-acetylated, acetylated at lysine 48 (AcLys48) or lysine 64 (AcLys64), were separated by HPLC and the chromatograms overlaid for comparison and reference. **C,** Recombinant human SIRT1 was incubated with AcLys48 peptide and NAD+ for evaluating the production of non-acetylated LKB1 peptide. **D,** Recombinant human SIRT1 was incubated with acetylated AcLys64 LKB1 peptide and NAD+ for evaluating the production of non-acetylated LKB1 peptide.
